# Systematic Comparison of Droplet‐Based and Microwell‐Based Platforms for Comprehensive Single‐Cell Transcriptomic Analysis in Clinical Samples

**DOI:** 10.1049/nbt2/9314222

**Published:** 2026-02-25

**Authors:** Shuai Wang, Yuxian Feng, Qiongdan Zhang, Yue Cui, Yi Qiao, Hao Huang, Xuan Pan, Jing Tu

**Affiliations:** ^1^ State Key Laboratory of Digital Medical Engineering, School of Biological Science and Medical Engineering, Southeast University, Nanjing, 210096, China, seu.edu.cn; ^2^ Department of Medical Oncology, Jiangsu Cancer Hospital & Jiangsu Institute of Cancer Research, The Affiliated Cancer Hospital of Nanjing Medical University, Nanjing, 210009, Jiangsu, China, njmu.edu.cn

**Keywords:** clinical samples, droplet-based platform, microwell-based platform, single-cell RNA sequencing, technical biases

## Abstract

Single‐cell RNA sequencing (scRNA‐seq) is widely utilized in tumor research. However, platform‐specific technical biases may impact data interpretation. This study compared the performance of droplet‐ and microwell‐based scRNA‐seq platforms in the analysis of clinical samples. Despite the similarities after batch effect correction, significant differences were observed in multiple aspects, including mRNA preference, cell type restoration, and gene expression patterns. The droplet‐based platform captured a higher proportion of immune cells, whereas the microwell‐based platform provided a more accurate immune cell representation. Differential gene expression, pseudotime, and cell–cell communication analyses further revealed platform‐dependent variations across multiple aspects. Overall, this study provides valuable insights into platform selection and optimization for cross‐platform data integration in single‐cell transcriptomics.

## 1. Introduction

Over the past decade, single‐cell RNA sequencing (scRNA‐seq) has undergone rapid advancements, evolving from early single‐cell digital gene expression profiling [1, 2] to methods capable of simultaneously analyzing thousands of transcripts per cell across tens of thousands of cells in a single experiment. The core principle underlying these techniques is the partitioning of cell suspensions into nanoliter‐scale reaction compartments to facilitate high‐throughput single‐cell transcriptomic profiling. Cells can be encapsulated in standard SBS plates, microwell arrays, or water‐in‐oil emulsions, followed by lysis and multiparameter analysis. The introduction of single‐cell barcoding strategies, including SMART‐seq [3], CEL‐seq [4], MARS‐seq [5], and SCRB‐seq, as well as their optimized versions, SMART‐seq2 [6], CEL‐seq2 [7], and mcSCRB‐seq [8], further refined transcriptomic barcoding and amplification, while reducing reliance on fluorescence‐activated cell sorting (FACS) for single‐cell isolation, thus streamlining experimental workflows.

Unlike traditional bulk RNA‐seq (bulk RNA‐seq), which captures the average gene expression levels of heterogeneous cell populations and thereby masks cellular heterogeneity, scRNA‐seq enables precise measurement of gene expression at the single‐cell level. This capability is critical for understanding the functional diversity and dynamic states of different cell types within complex tissues, particularly in highly heterogeneous diseases such as cancer [9]. By leveraging scRNA‐seq, researchers can distinguish tumor cells, immune cells, stromal cells, and other cellular subpopulations, thereby providing deeper insights into their distinct roles in disease progression.

In recent years, the application of microfluidic technology has driven further innovation in scRNA‐seq, significantly increasing cell throughput while reducing costs. Droplet‐based methods such as Drop‐seq [10], inDrop [11], and 10x Genomics Chromium [12] employ microfluidic droplet encapsulation to isolate single cells, with barcoded beads labeling transcripts for downstream sequencing. Similarly, high‐density microwell‐based systems incorporate barcoded beads in a format akin to droplet‐based platforms, exemplified by Seq‐Well [13], Microwell‐seq [14], and commercially available systems such as Singleron GEXSCOPE [15] and BD Rhapsody.

Drop‐seq and Microwell‐seq offer distinct advantages in experimental workflow and cell capture efficiency. Drop‐seq, based on microfluidic technology, encapsulates individual cells with barcoded beads within oil‐phase droplets, followed by lysis, reverse transcription, and sequencing. This approach supports extremely high throughput [16–18], making it well suited for analyzing vast cellular populations and particularly efficient at capturing immune cells (e.g., T cells and B cells), making it an excellent choice for immune system studies and profiling rare subpopulations. In contrast, Microwell‐seq employs microwell arrays, allowing single cells to settle into wells along with barcoded beads via gravity or centrifugation. This physical isolation approach offers a unique advantage in capturing larger or morphologically distinct cells [19, 20], such as neutrophils, making it particularly valuable for studying myeloid cells, inflammatory responses, and hematopoiesis. Furthermore, Microwell‐seq requires minimal specialized equipment, making it a cost‐effective choice for large‐scale single‐cell atlas construction [21].

Although high‐throughput scRNA‐seq technologies generate seemingly comparable datasets [22, 23] and are often used interchangeably in different biological contexts, their underlying methodologies introduce platform‐specific biases, detection preferences, and technical constraints. These inherent differences influence their performance across various cell types and tissue environments, rendering certain platforms uniquely suited for specific biological applications. While scRNA‐seq technologies are advancing RNA sequencing and research, as noted in several studies, each platform has its own strengths and limitations [24–27]. However, systematic comparative studies evaluating high‐throughput scRNA‐seq platforms remain relatively scarce, with most assessments relying on homogeneous cell cultures or artificially mixed cell pools [24]. Such experimental designs fail to fully capture the true performance of different platforms in detecting cellular heterogeneity and delineating cell lineage identities within complex tissue samples.

In this study, we conducted a systematic comparison of droplet‐ and microwell‐based scRNA‐seq technologies using lung adenocarcinoma samples derived from the same patients. Our analysis comprehensively evaluates multiple aspects, including highly variable gene (HVGs) selection, cell type annotation, copy number variation (CNV) inference, gene expression characteristics within identical cell types, and immune cell composition. The findings provide critical insights for optimizing scRNA‐seq experimental design, facilitating cross‐platform data interpretation, and enabling robust integration of multi‐platform scRNA‐seq datasets.

## 2. Results

### 2.1. Platform‐Specific Preferences in Data Quality and Read Distribution

Single‐cell suspensions were prepared from freshly resected tumor tissues collected from the same anatomical site of each patient. For each patient, the tissue was evenly divided into two halves and processed in parallel under identical experimental conditions for the droplet‐ and microwell‐based platforms to minimize sampling and technical variability. Using platform‐specific pipelines, we obtained a total of 47,401 high‐quality cells from the droplet‐based platform, comprising 21,922, 10,784, and 14,795 cells from the three respective samples. In parallel, the microwell‐based platform yielded 33,915 high‐quality cells, including 12,231, 8832, and 12,952 cells from each sample, respectively.

The two platforms exhibited notable differences in quality control (QC) metrics. We observed that the droplet‐based platform typically yielded higher total read counts and gene detection per cell compared to the microwell‐based platform (Figure 1A,B). One plausible explanation for this observation is the difference in mRNA capture efficiency between the two platforms. Droplet‐based platforms perform cell lysis and reverse transcription within physically confined microdroplets, where a high local concentration of barcoded oligo (dT) primers facilitates rapid and efficient capture of polyadenylated transcripts. This confined reaction environment reduces RNA diffusion and loss, resulting in higher numbers of captured transcripts and consequently increased read counts and gene detection per cell relative to microwell‐based approaches. After rigorous QC, 43,493 cells sequenced using the droplet‐based platform and 31,807 cells sequenced using the microwell‐based platform were retained for downstream analysis.

Figure 1A comparative overview of data quality and gene expression characteristics across two single‐cell sequencing platforms. (A,B) nFeature and nCount are quality metrics in filtered cells derived from two platforms. (C) Proportion of reads mapped to genomic regions in six samples. (D) %MT quality metrics in filtered cells derived from two platforms. (E) Expression of stress‐related transcripts in droplet‐ and microwell‐based platform data generated from six samples.

**Figure:**
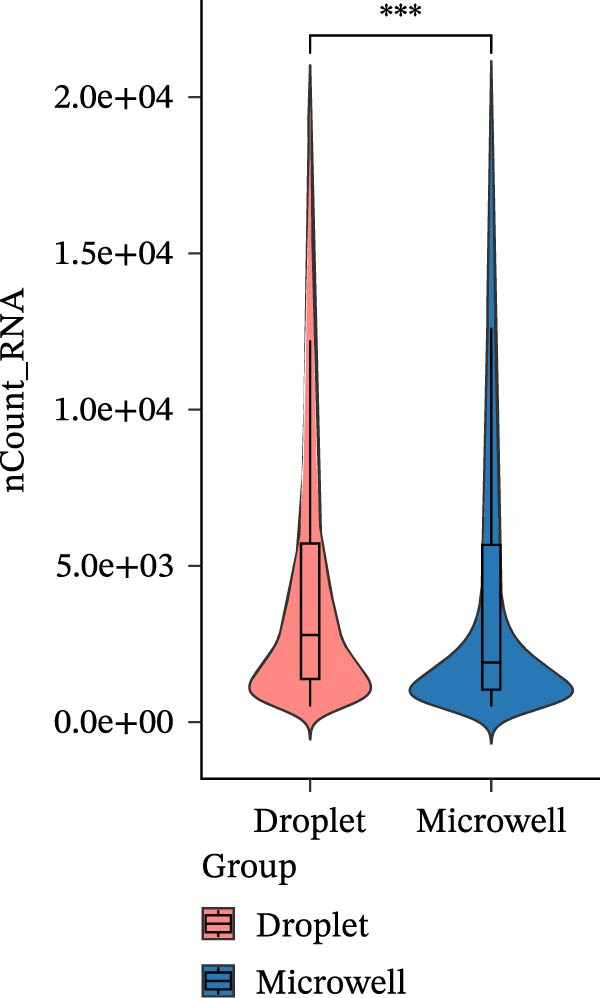
(A)

**Figure:**
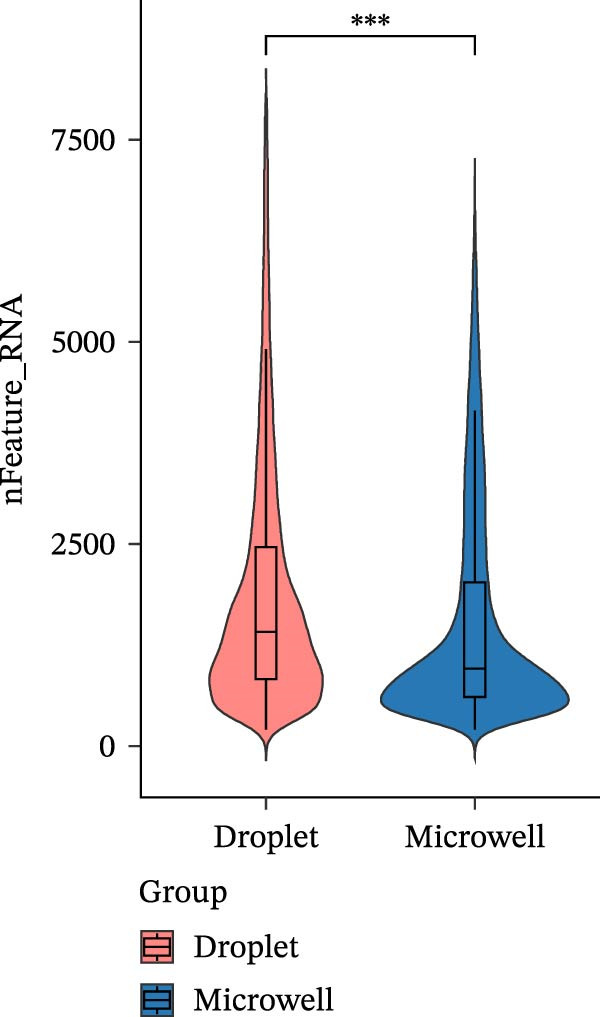
(B)

**Figure:**
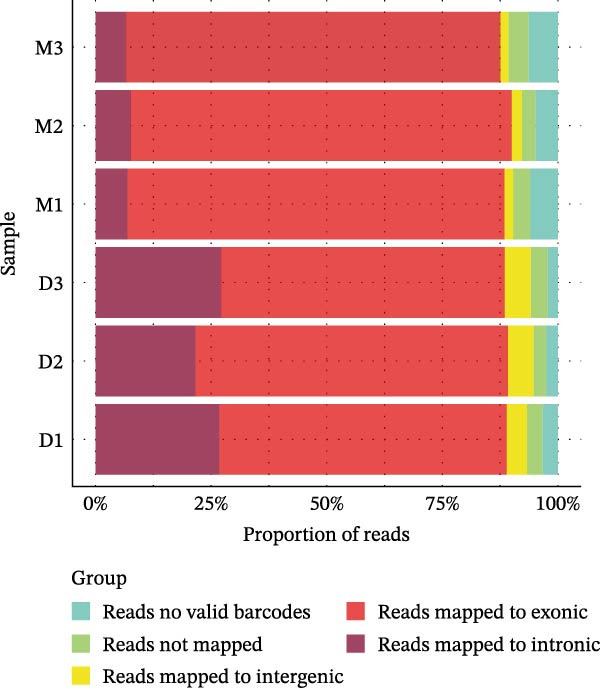
(C)

**Figure:**
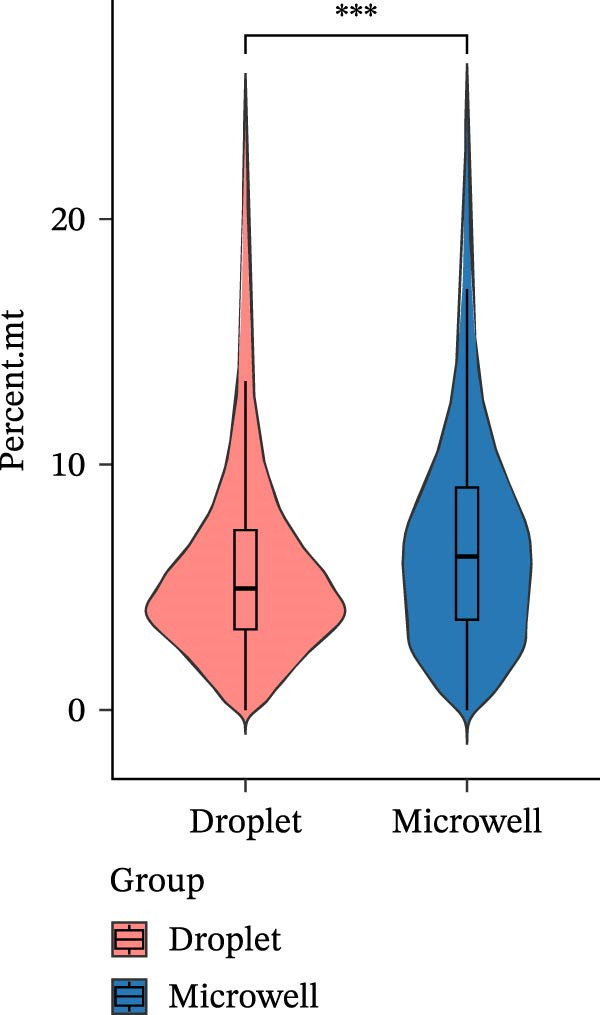
(D)

**Figure:**
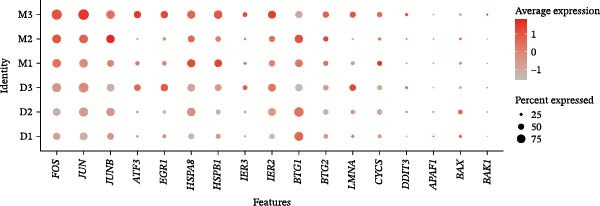
(E)

Although the microwell‐based platform detected fewer transcripts and genes per cell compared to the droplet‐based platform, it conserved a higher proportion of mature mRNA. On the droplet‐based platform, the mean proportion of confidently mapped reads to the exon regions stood at 62.93% of the total, while the intron regions captured an average of 24.93% of the total reliably mapped reads. In contrast, the microwell‐based platform exhibited a markedly different profile. Here, the average proportion of reliably mapped reads to the exon regions reached 87.62% of the total, with a mere 7.58% of the total reliably mapped reads being attributed to the intron regions (Figure 1C). Moreover, a high ratio of mitochondrial DNA‐encoded genes (%MT) mapping was observed in the microwell‐based platform (Figure 1D), which is commonly regarded as a positive indicator of cellular stress, apoptosis, and low‐quality cells [28, 29]. Also, stress‐associated transcripts such as FOS, JUN, and HSPA8 exhibited higher expression levels in the microwell‐based platform, despite no significant differences being observed in the overall expression of stress‐related transcripts (Figure 1E), suggesting potential platform‐specific preference of stress‐related cellular features.

To validate the consistency of these findings and rule out potential sample‐specific biases, we further retrieved publicly available datasets generated by both the droplet‐ [30] and microwell‐based platforms [31]. After a meticulous and comprehensive comparative analysis of these external datasets, we found that the mapping proportions were strikingly consistent with our own experimental results.

### 2.2. Evaluating and Reducing Batch Effects Between Platforms

During scRNA‐seq, batch effects can arise in the sequencing process. In the PC analysis (PCA) analysis (Figure S1A), samples from the two platforms were clearly distinguished along the first principal component (PC), while biological variability of the three samples existed. These results indicate that the batch effects between platforms may outweigh the biological differences between samples from the same tissue type. We visualized the clustering patterns of samples from both platforms using UMAP to assess intraplatform variability and employed two clustering evaluation metrics to quantify the magnitude of batch effects. As shown in Figure 2A,B, the UMAP plots of both platforms exhibit certain similarities. Notably, Sample2 and Sample3 form distinct, isolated regions in the plot, which may reflect the specificity of these samples compared to others. Additionally, the UMAP plots reveal overlapping areas between the three sample groups, suggesting some degree of homogeneity among certain cell populations in the lung adenocarcinoma samples. Specific evaluation metrics indicate that the Batch_ARI value for the droplet‐based platform (D1–D3) was 0.9048, higher than that of the microwell‐based platform (M1–M3) at 0.7635. Similarly, the Batch_ASW value for the droplet‐based platform was 0.4757, slightly higher than the 0.4318 observed for the microwell‐based platform (Figure 2A, B).

Figure 2Integration and visualization of single‐cell data across platforms. (A,B) UMAP plot of the three droplet‐ platform and microwell‐based platform samples, colored by sample identity. Platform labels in the legend are abbreviated as “M” for droplet‐based and “D” for microwell‐based. (C) UMAP plot of the six samples from two platforms, colored by sample identity. (D–F) UMAP plots of six samples from two platforms after correction using Seurat, Harmony, and Liger software.

**Figure:**
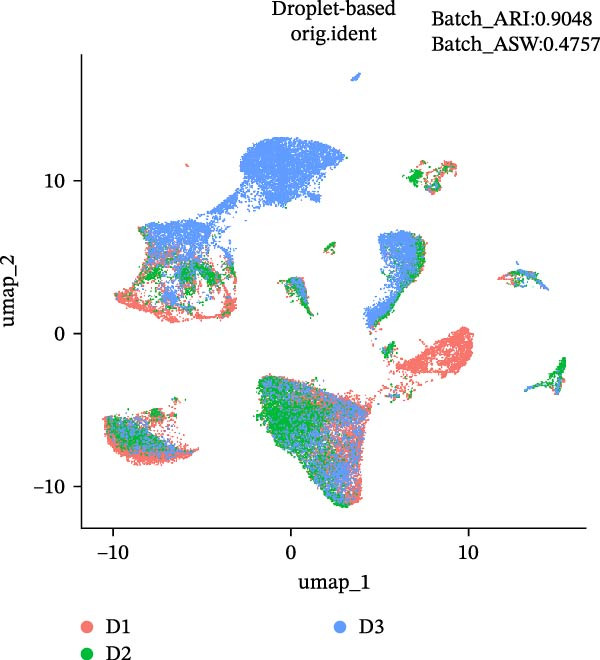
(A)

**Figure:**
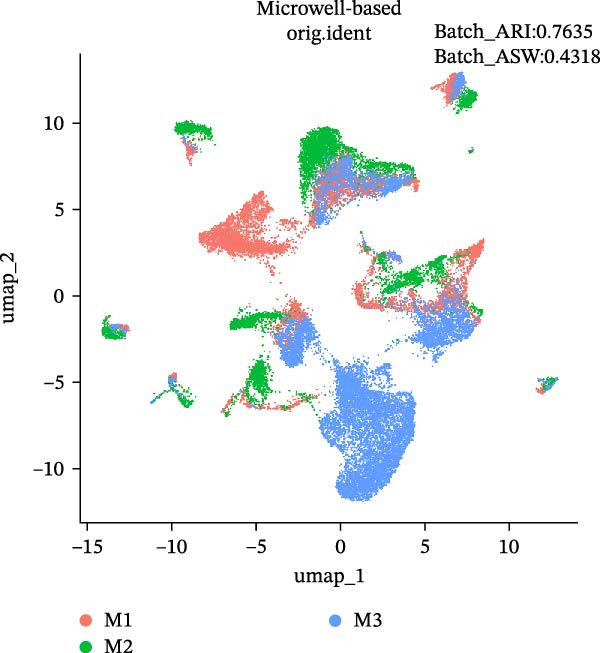
(B)

**Figure:**
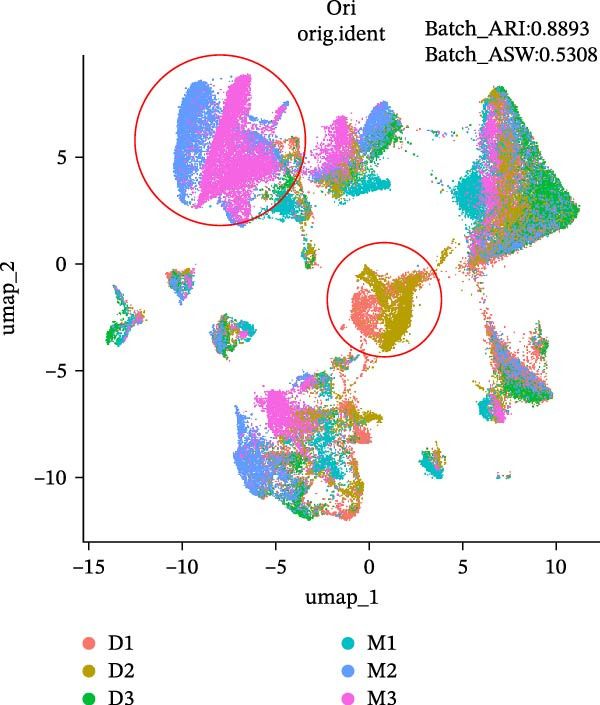
(C)

**Figure:**
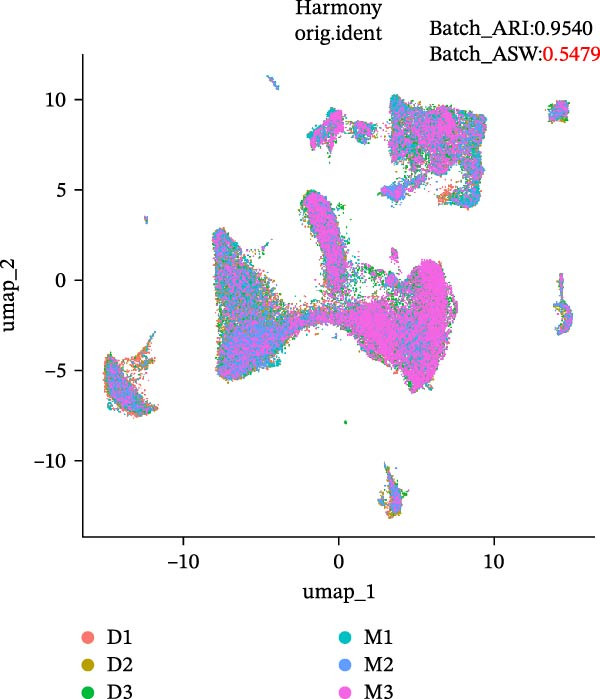
(D)

**Figure:**
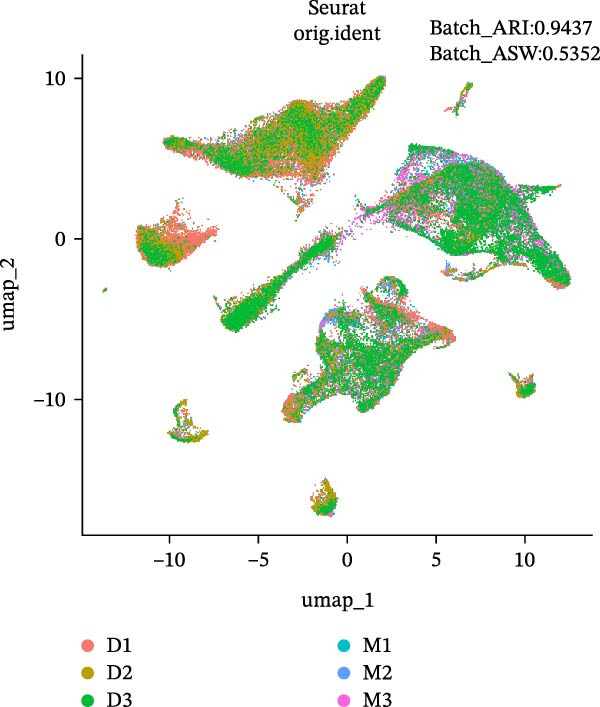
(E)

**Figure:**
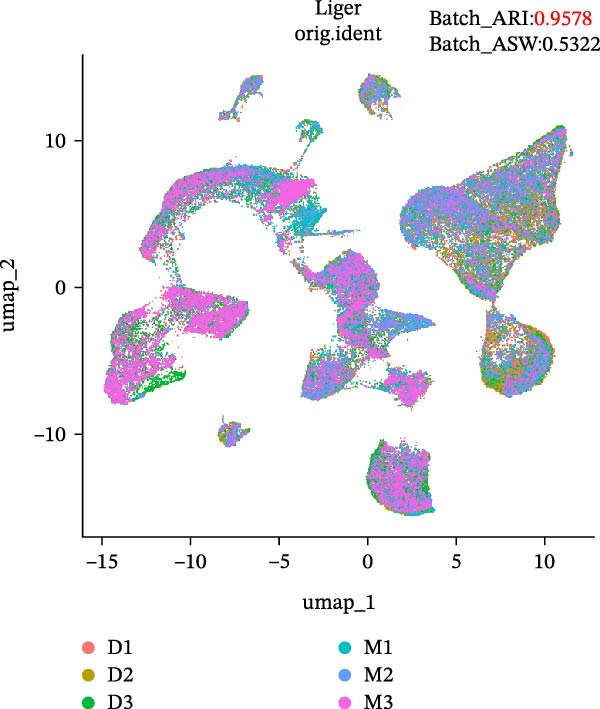
(F)

We further integrated the six samples for de‐batch‐effect analysis. Although the samples from the same source tended to cluster together (areas highlighted by red circles in Figure 2C), suggesting good biological replicability of these cells, a large number of single cells still suffered from significant batch effects. To assess the effectiveness of mainstream single‐cell transcriptomics analysis tools in mitigating batch effects arising from two different platforms, we performed batch effect correction on the six samples. UMAP clustering revealed residual batch effects due to platform differences and individual variability, with the uncorrected data yielding Batch_ARI and Batch_ASW values of 0.8893 and 0.5308, respectively (Figure 2C). We then evaluated several advanced batch correction methods, including Harmony, Seurat, and Liger, which have been validated in a recent study [32]. Postcorrection UMAP visualizations demonstrated effective mixing of cells across samples for all methods, and quantitative metrics improved significantly, with Batch_ARI values of 0.9540 (Harmony, Figure 2D), 0.9437 (Seurat, Figure 2E), and 0.9578 (Liger, Figure 2F), as well as Batch_ASW values of 0.5479 (Harmony), 0.5352 (Seurat), and 0.5322 (Liger).

### 2.3. Microwell‐Based Platform Accurately Restored Immune Cell Proportion

To evaluate the distribution characteristics of preferences on cell types of the two platforms after batch effect correction, cell clusters were classified into epithelial cells, immune cells, and stromal cells, with the latter primarily representing endothelial and mesenchymal cells. Immune cells constitute the predominant population, while epithelial cells exhibit a more localized distribution. In contrast, stromal cells are relatively sparse and are primarily located at the periphery (Figure 3A, B). A dot plot of marker gene expression revealed distinct expression patterns among the three major cell populations derived from the droplet‐based (Figure S2A–C) and microwell‐based platforms (Figure S2B–D), supporting the classification validity.

Figure 3Annotation of major cell types and the comparison of immune cell proportions across platforms. (A,B) Batch‐corrected UMAP plot of the three droplet‐based platform/microwell‐based platform samples, colored by three major cell types. (C) Column stacking chart of cell types for each sample. (D) Bar chart of immune cell ratio on two platforms. (E,F) Batch‐corrected UMAP plot of the three droplet‐based platform/microwell‐based platform samples, colored by cell type. (G) Bar plot showing the average number of detected genes (nfeature_RNA) per cell type across two platforms.

**Figure:**
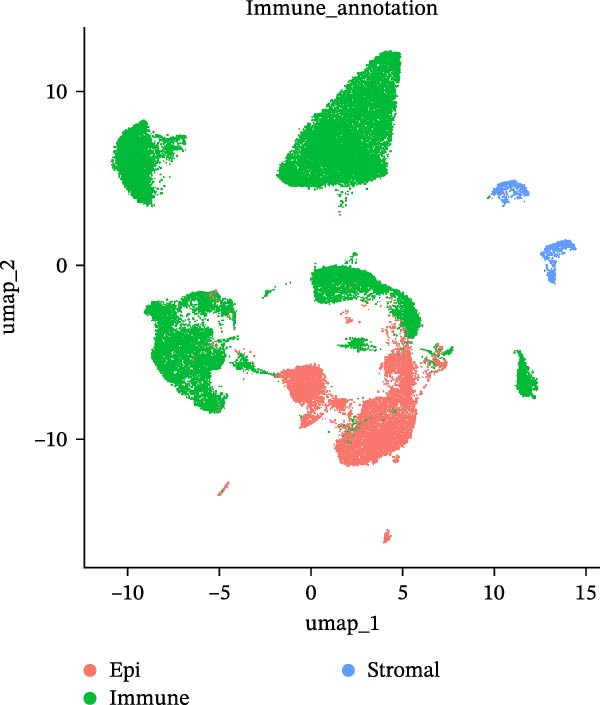
(A)

**Figure:**
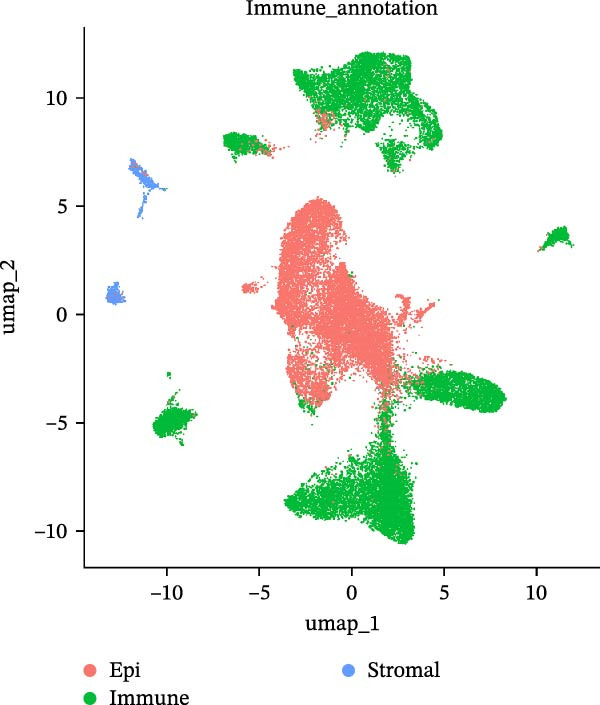
(B)

**Figure:**
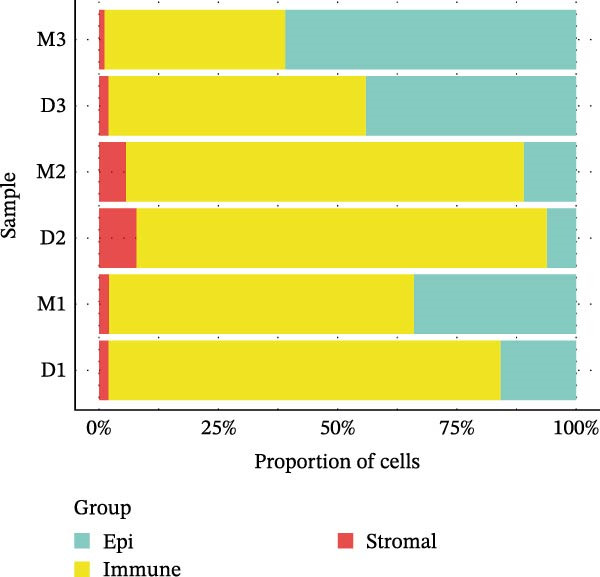
(C)

**Figure:**
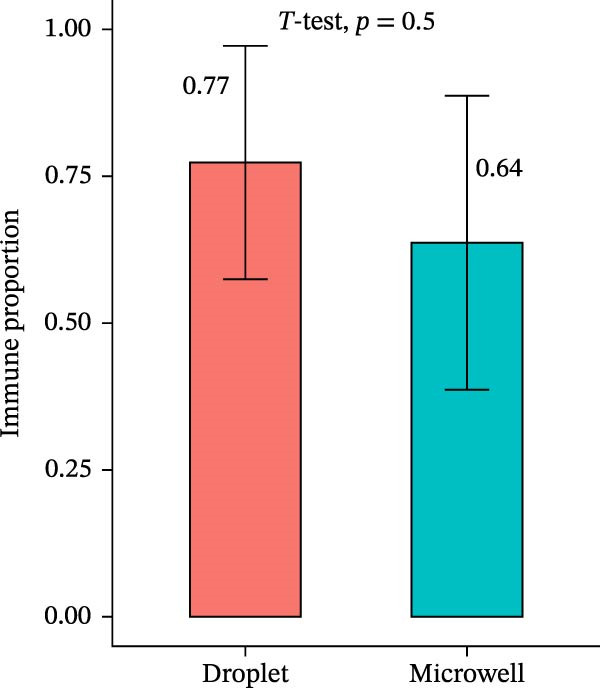
(D)

**Figure:**
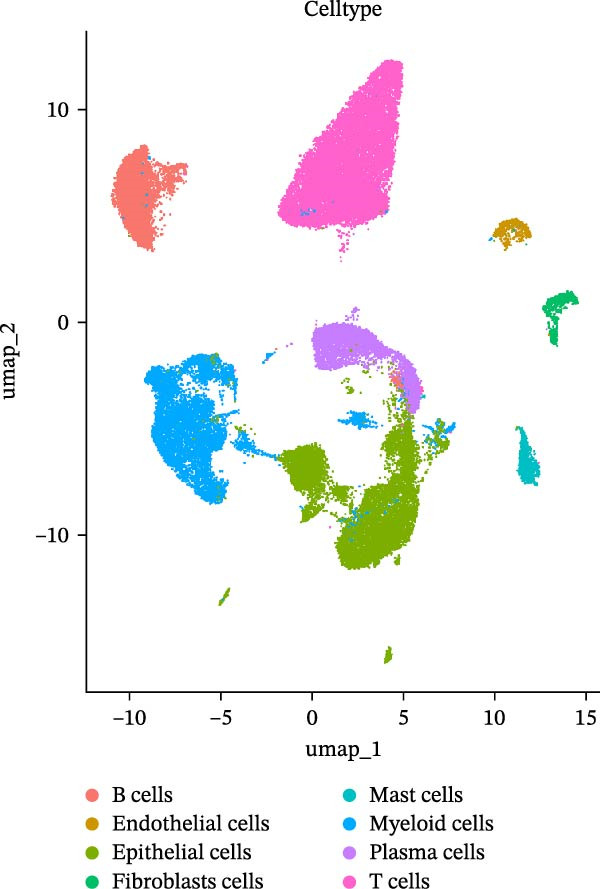
(E)

**Figure:**
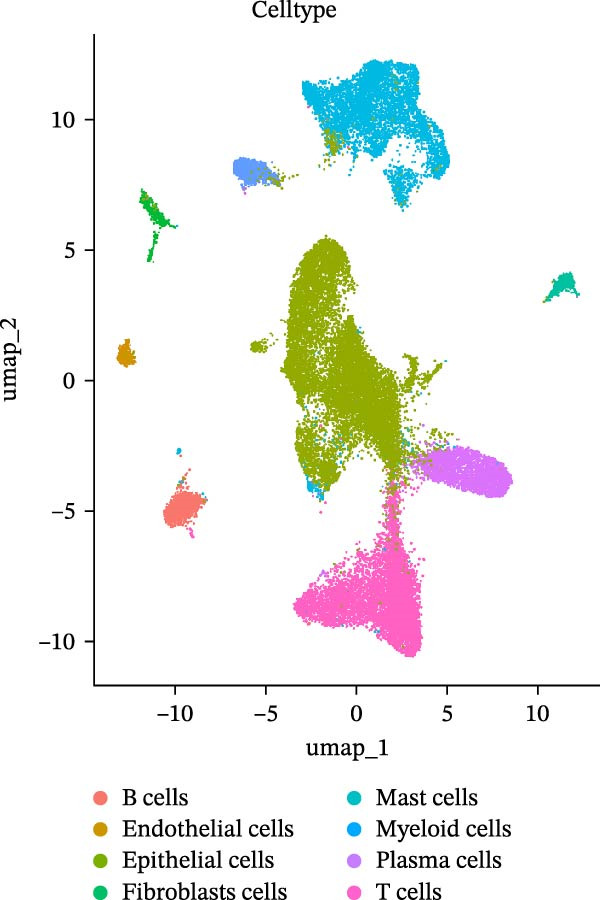
(F)

**Figure:**
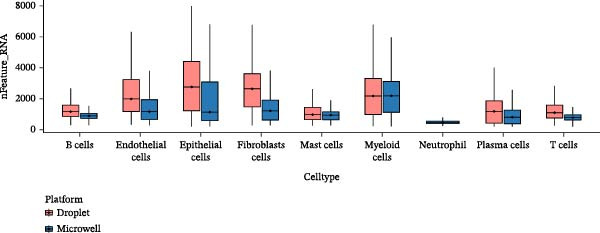
(G)

The proportion of the three major cell populations differs across the two platforms (Figure 3C). In each sample, the proportion of immune cells on the droplet‐based platform is greater than that on the microwell‐based platform. The *t*‐test of the immune cell ratio between the two platforms (Figure 3D) showed that although there was no significant difference in the proportion of captured immune cells between the samples, the mean proportion of immune cells on the microwell‐based platform was closer to the flow cytometry results we conducted (Figure S3A,B). One possible explanation is that microwell‐based platforms capture cells through passive settling into microwells followed by lysis, in contrast to the active microfluidic droplet encapsulation used in droplet‐based platforms. This passive capture strategy is likely more reflective of the true tissue composition. Because immune cells are naturally suspended, they are less efficiently captured by passive settling compared with droplet encapsulation, which preferentially captures small, freely suspended cells, resulting in a lower representation of immune cells in microwell‐based datasets. Moreover, several studies have demonstrated that droplet‐based platforms tend to overestimate immune cell abundance [32–34].

### 2.4. Platform‐Dependent Variability in Cell Subtype Detection

To further explore the platform‐specific differences in cellular composition, resolution, and functional characterization, we conducted a comprehensive comparison between the droplet‐ and microwell‐based scRNA‐seq datasets.

First, the droplet‐based dataset yielded eight cell subtypes (Figure 3E), whereas the microwell‐based dataset yielded nine (Figure 3F). Notably, the expression of neutrophil marker genes, including *FCGR3B, CXCR2*, and *CSF3R* [35], was nearly undetectable in any cluster within the droplet‐based dataset (Figure S4A–C). In contrast, a distinct neutrophil population was exclusively identified in the microwell‐based dataset (Figure S4D–F). Furthermore, the median number of genes detected per cell was lowest in neutrophils compared to the other cell types (Figure 3G), suggesting that microwell‐based platforms exhibit higher capture efficiency for fragile cells with low mRNA content than droplet‐based systems. This finding is consistent with previous studies [36, 37]. This is likely due to the high RNase content in neutrophil granules, their fragility under microfluidic pressure, and potential RNase‐mediated RNA degradation in droplet‐based datasets, whereas microwell‐based datasets may better preserve RNA through stronger RNase inhibition and bead washes [38].

Refer to [39], we assessed whether platform‐specific discrepancies exist in gene expression patterns within the same cell types. For each target cell type, differential expression analysis was performed using the remaining cell types as a reference. A subset of genes exhibited opposite log fold change (logFC) signs between the droplet‐ and microwell‐based platforms, indicating conflicting expression trends. These genes were subsequently visualized in a heatmap to highlight the differences. (Figure 4A and Figure S5A–D and Figure S6A–C). One possible hypothesis is that RNAs susceptible to degradation are preferentially lost during the microwell‐based protocol. However, mRNA stability is influenced by multiple factors, and current literature provides no direct evidence that the specific transcripts exhibiting opposite expression patterns between the two platforms are intrinsically unstable or prone to rapid degradation [39].

Figure 4Heterogeneity analysis of cell types between platforms. (A) Heatmap of genes with opposite expression patterns in fibroblasts on two platforms. (B, D, F) Number of inferred cell–cell interactions based on droplet‐based scRNA‐seq data. (C, E, G) Number of inferred cell–cell interactions based on microwell‐based scRNA‐seq data. (H) Bar plot showing the total number of inferred cell–cell interactions in each individual sample. (I) Ligand–receptor interaction bubble plot for patient 1.

**Figure:**
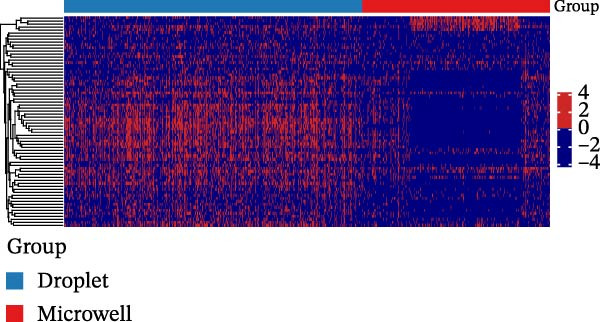
(A)

**Figure:**
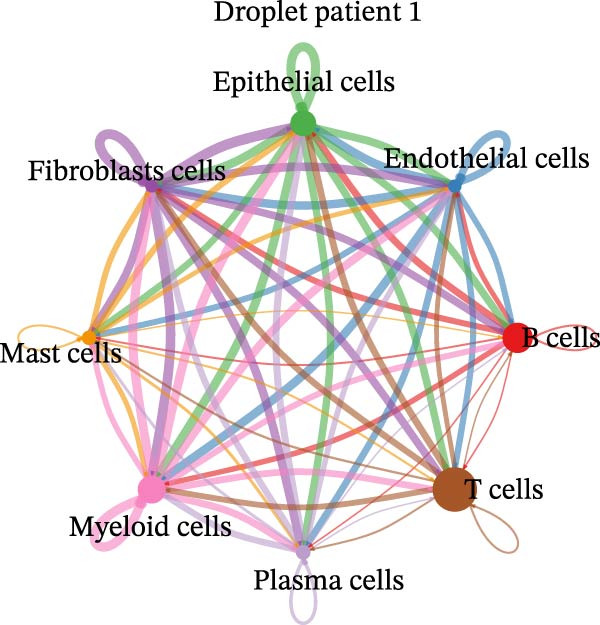
(B)

**Figure:**
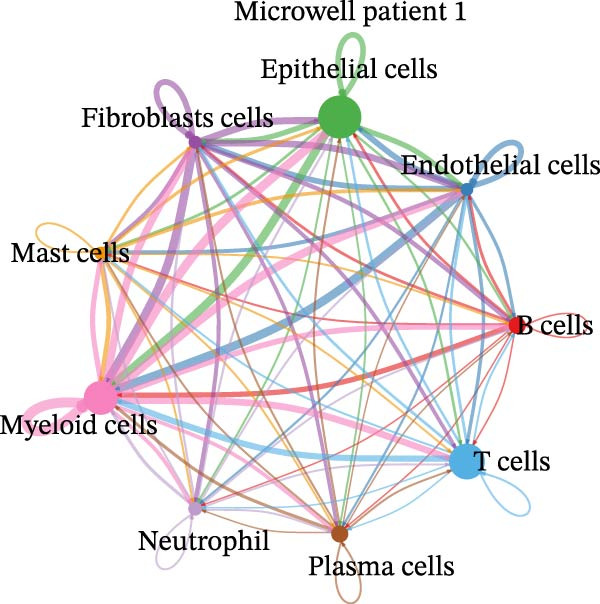
(C)

**Figure:**
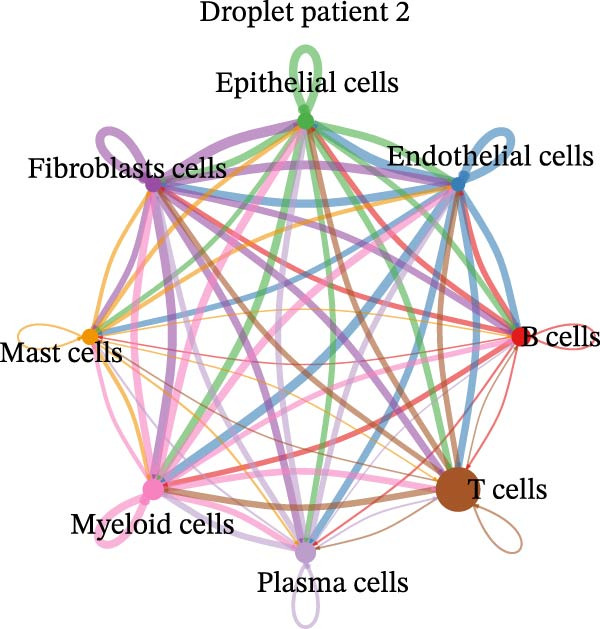
(D)

**Figure:**
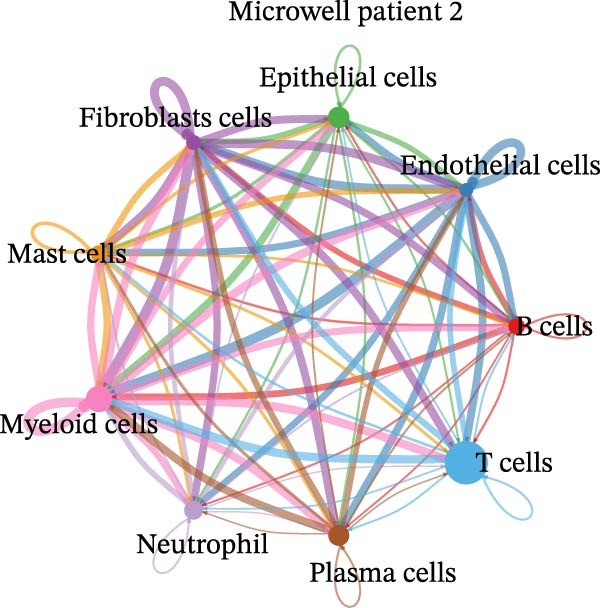
(E)

**Figure:**
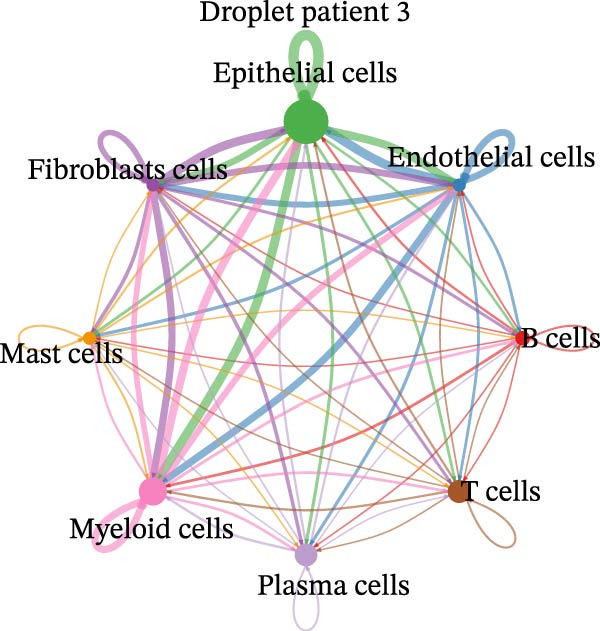
(F)

**Figure:**
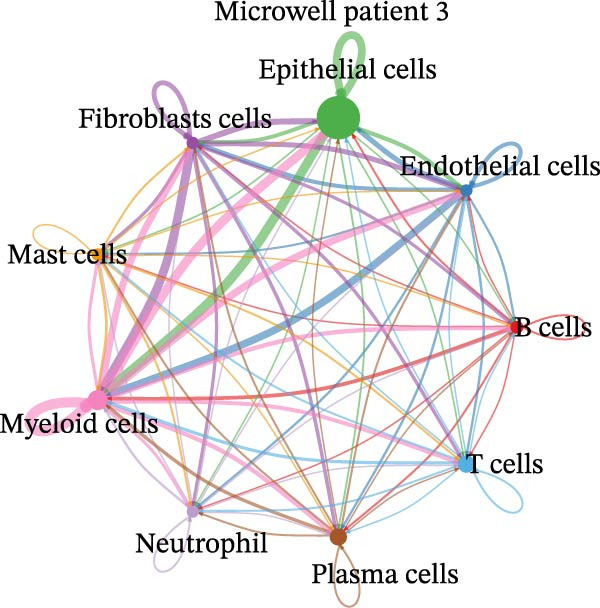
(G)

**Figure:**
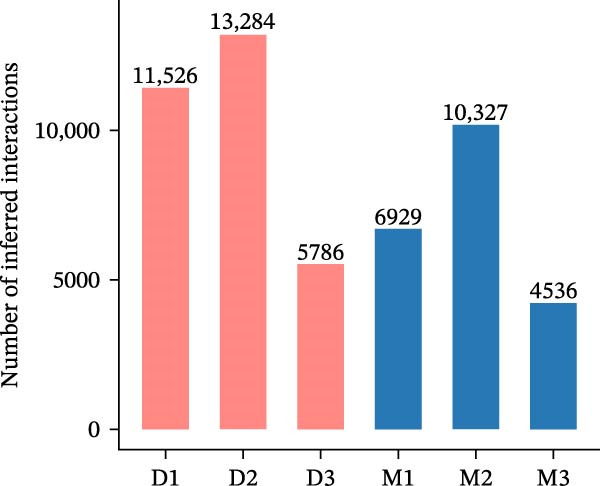
(H)

**Figure:**
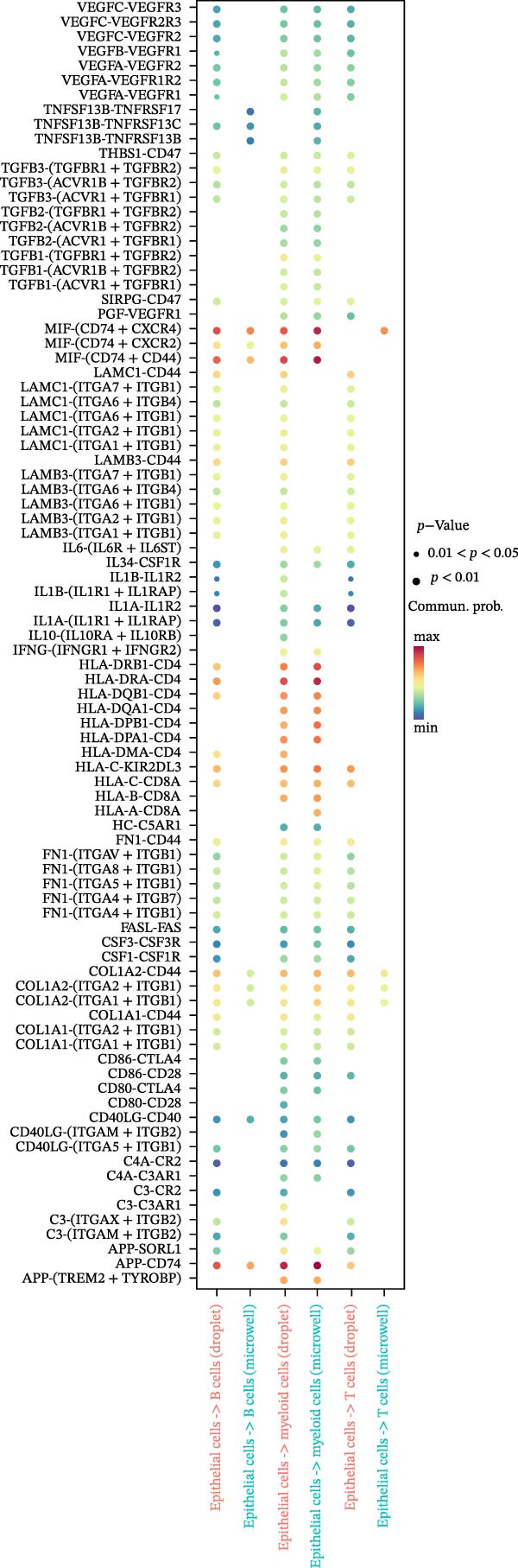
(I)

As for the cell–cell communication patterns, we performed cell–cell interaction analyses for each of the three patients separately on the droplet and microwell platforms. In Patient 1, the Droplet‐based dataset exhibited a markedly higher number of inferred cell–cell interactions than the Microwell‐based dataset, and the corresponding interaction network showed more extensive intercellular connections across multiple cell types (Figure 4B, C), whereas interactions inferred from the Microwell platform were more restricted and predominantly centered around myeloid cells. In contrast, Patients 2 and 3 displayed comparable numbers of inferred cell–cell interactions between the two platforms (Figure 4D–G), suggesting that platform‐related differences in cell–cell communication may vary across individuals. To further quantify these observations, we summarized the total number of inferred cell–cell interactions for each individual sample using bar plots (Figure 4H), which revealed that, across all patients, samples generated using the Droplet platform consistently exhibited higher total interaction numbers than their Microwell counterparts. This discrepancy may be partially attributable to differences in capture efficiency between the two platforms, as the Droplet platform generally captures a larger number of cells and transcripts, thereby increasing the likelihood of detecting ligand–receptor (LR) interactions. Finally, LR interaction analysis focused on patient 1 demonstrated that, in several key immune‐related signaling pathways, including MHC, IL1, TNF, SPP1, MIF, TGFβ, and more, the droplet‐based data captured a more comprehensive spectrum of interactions (Figure 4I), which facilitates a more detailed characterization of the tumor immune microenvironment, while Patients 2 and 3 (Figure S7A, B) showed a consistent trend, supporting the robustness of these observations.

As previously described, the droplet‐based platform demonstrated higher sensitivity in single‐cell capture compared to the microwell‐based platform, enabling the enrichment of a greater number of immune cells. Based on canonical marker gene expression, 5 T cell subtypes were identified in the droplet‐based dataset (Figure 5A, including cytotoxic CD8^+^ effector T cells, immunosuppressive CD4^+^ T cells, naïve T cells, natural killer T (NKT) cells, and regulatory T cells (Tregs). In contrast, the microwell‐based platform detected only four of these subtypes (Figure 5B), lacking the immunosuppressive CD4^+^ T cell population. Although both platforms used tissue samples from the same source, and consistently identified the major T cell subtypes, with naïve T cells representing the most abundant population in both datasets (Figure S8), the absence of immunosuppressive CD4^+^ T cells in the microwell‐based data indicates its relatively limited sensitivity in detecting rare immune cell subsets.

Figure 5(A,B) UMAP plot of the droplet‐based platform/microwell‐based platform T cells, colored by subcell type. (C,D) UMAP plot of the droplet‐based platform/microwell‐based platform epithelial cells, colored by clusters. (E,F) Visualization of cell states inferred from pseudotime analysis on the droplet‐based platform/microwell‐based platform, showing the distribution of cells across different developmental states.

**Figure:**
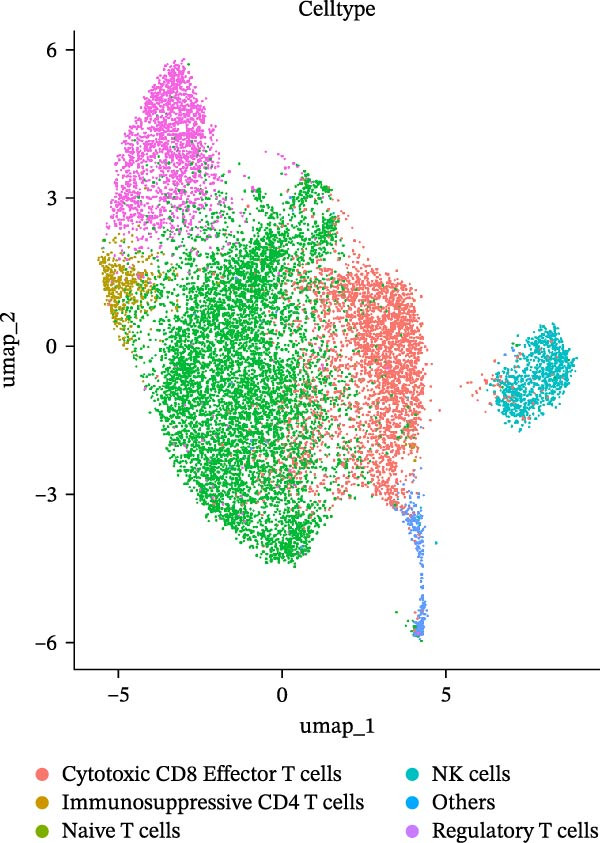
(A)

**Figure:**
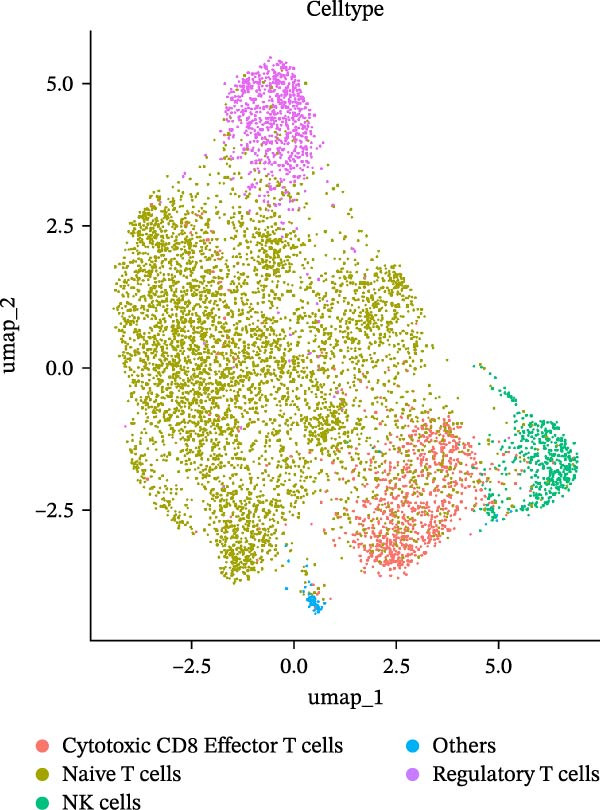
(B)

**Figure:**
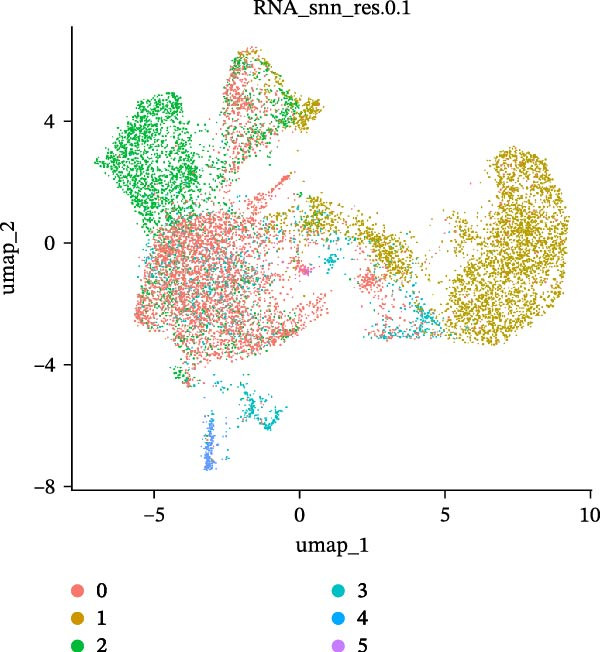
(C)

**Figure:**
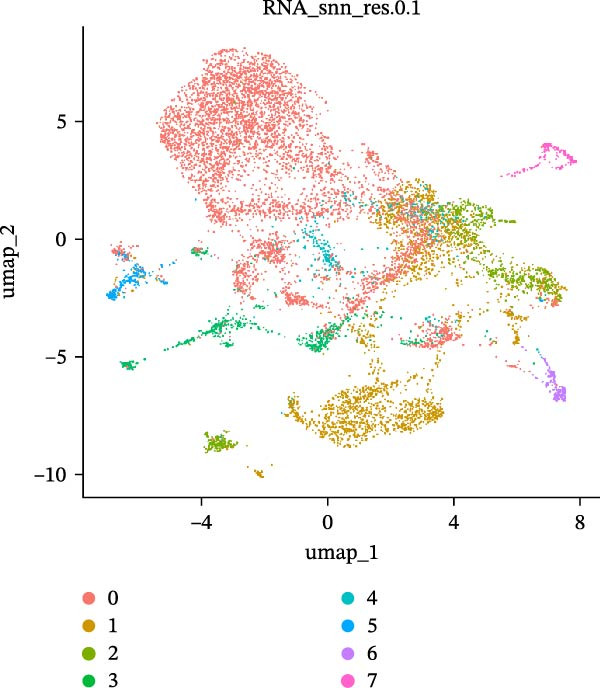
(D)

**Figure:**
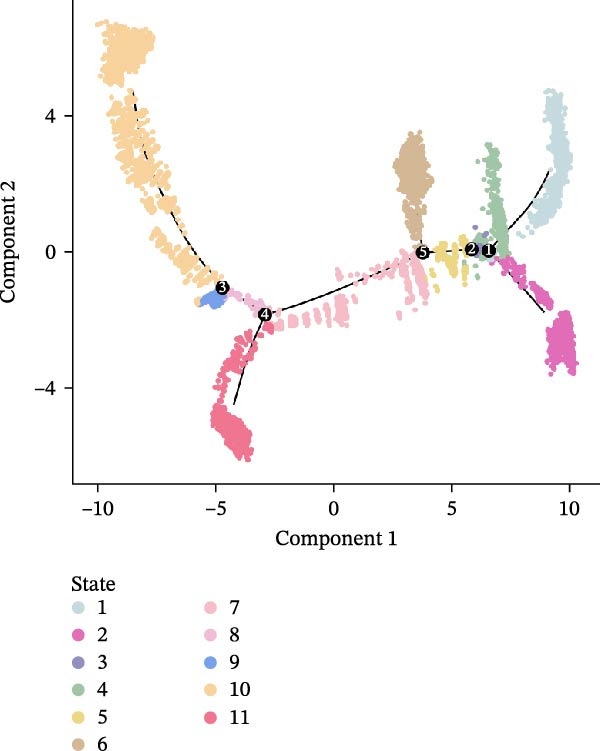
(E)

**Figure:**
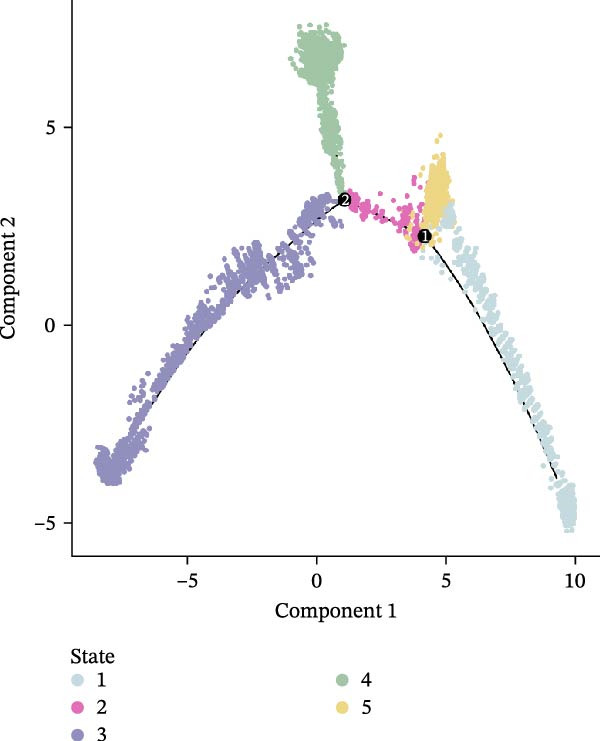
(F)

Further subcluster analysis of epithelial cells revealed that the microwell‐based platform identified six clusters, whereas the droplet‐based platform identified eight clusters (Figure 5C, D). UMAP visualization indicated that the microwell‐based platform exhibited more distinct clustering, suggesting differences in cellular resolution between the platforms.

To investigate the potential heterogeneity introduced by different sequencing platforms in analyzing tumor progression and differentiation trajectories, we performed pseudotime analysis on epithelial cell subclusters (Figure 5E, F). The results revealed that the droplet‐based platform exhibited 11 cellular states, whereas the microwell‐based platform exhibited only five states. Furthermore, the droplet‐based platform displayed a greater number of branch points, indicating a more complex evolutionary trajectory of cancer cells. In contrast, the fewer states detected in the microwell‐based platform did not distinguish certain critical cellular states.

## 3. Discussion

This study compares the performance of two scRNA‐seq technologies, droplet‐ and microwell‐based, in clinical samples. The results reveal significant technical and biological differences between the two platforms, which have critical implications for interpreting single‐cell transcriptomic data and advancing tumor biology research. Selecting an appropriate sequencing platform is crucial, as it directly affects the reliability of single‐cell expression profiles and influences both time and cost considerations. Researchers must carefully evaluate the characteristics of different platforms to select the most suitable technology for their scientific inquiries, ensuring the accuracy and reliability of the data.

One of the most striking findings is the difference in read mapping patterns between the two platforms. The droplet‐based platform exhibits a higher proportion of reads mapped to intronic regions (24.93%), suggesting a lower efficiency in capturing exonic sequences compared to the microwell‐based platform, which achieves an exonic mapping rate of 87.62%. These discrepancies in mapping efficiency may reflect variations in sequencing depth, RNA capture strategies, and platform‐specific biases. Notably, similar mapping patterns were observed when analyzing publicly available datasets, further supporting the notion that these differences originate from the sequencing platforms rather than sample‐specific biases.

Our analysis identified significant platform‐specific technical biases between the datasets. PCA reveals a clear spatial separation between the two platforms, suggesting that technical differences may overshadow the biological variations among samples. QC metrics further highlight platform‐specific discrepancies, with the droplet‐based platform demonstrating higher gene detection sensitivity and a lower proportion of mitochondrial transcripts compared to the microwell‐based platform. Clustering evaluation metrics indicate that batch effects are more pronounced in the microwell‐based platform, emphasizing the impact of sequencing technology bias on batch effect magnitude. Further integration and clustering analysis, along with batch effect correction tools such as Harmony, Seurat, and Liger, demonstrate their effectiveness in mitigating cross‐platform batch effects. These findings underscore the importance of accounting for sequencing platform biases and batch effects when integrating single‐cell datasets from multiple technologies.

Additionally, we assess the differences in cell composition and marker gene expression between the two platforms. The droplet‐based platform detects a higher proportion of immune cells, consistent with previous studies suggesting that droplet platforms may preferentially capture nonadherent, suspension‐growing immune cells. In contrast, the microwell‐based platform provides a more accurate immune cell proportion, aligning with flow cytometry results. These discrepancies highlight the importance of selecting an appropriate sequencing platform when studying specific cell populations in the tumor microenvironment.

Our tumor microenvironment analysis further reveals differences in cell type and subtype detection between the two platforms. The droplet‐based platform identifies a broader range of immune cell subpopulations, whereas the microwell‐based platform demonstrates superior detection of neutrophils, a cell type almost absent in the droplet‐based dataset. These differences suggest that platform‐specific biases may affect the interpretation of tumor immune microenvironments, particularly in terms of immune cell composition and function. Beyond cell composition and gene expression differences, we also observe inconsistencies in gene expression trends between the two platforms. Such discrepancies may arise from differences in library preparation methods or the sensitivity of detecting low‐abundance transcripts. Furthermore, CellChat analysis reveals distinct cell–cell communication patterns between the two platforms. The microwell‐based platform uniquely detects neutrophils, and their potential role in the tumor immune microenvironment. LR analysis indicated that key signaling pathways were more comprehensively detected on the droplet‐based platform. Despite technical biases, both platforms identify strong intercellular communication signals, particularly among epithelial cells, myeloid cells, and B cells, which may represent core interaction patterns within the lung adenocarcinoma microenvironment. Finally, pseudotime analysis further indicates that the droplet‐based platform exhibits more complex developmental trajectories, including additional branching points, suggesting potential differences in their ability to resolve tumor progression and development.

In conclusion, this study emphasizes the importance of carefully considering platform‐specific characteristics in scRNA‐seq experiments. The choice of sequencing platform not only impacts data quality but also significantly influences cell type identification, subpopulation analysis, and gene expression profiling accuracy. We uncover systematic differences between the droplet‐ and microwell‐based platforms in read mapping, cell composition, cell–cell communication, gene expression patterns, and biological pathway enrichment. These differences may stem from library construction strategies, sequencing depth, RNA capture efficiency, and data processing pipelines. Notably, platform‐specific differences were particularly evident in immune cell populations. The droplet‐based platform consistently captured a larger number of immune cells, making it advantageous for studies focusing on immune composition or immune‐tumor interactions. In contrast, the microwell‐based platform showed improved recovery of neutrophils, a cell type known to be fragile and susceptible to loss during dissociation and processing. This suggests that certain observed differences in immune cell representation across platforms may primarily reflect technical preferences rather than underlying biological variation. Therefore, for researchers with a specific interest in immune cells or neutrophils, platform choice should be carefully considered, as it may substantially influence cell‐type abundance and downstream biological interpretations. Our findings provide critical insights for cross‐platform data integration and highlight the necessity of correcting and optimizing for platform‐specific biases when conducting single‐cell studies. Future research should further explore the underlying mechanisms driving platform‐specific variations and evaluate their impact on scRNA‐seq data analysis. Moreover, advancements in scRNA‐seq methodologies and experimental workflows, such as improving sensitivity for low‐abundance transcripts, developing more robust cross‐platform batch effect correction methods, and integrating multi‐omics approaches, will enhance data comparability and improve the accuracy of biological interpretations. These efforts will not only advance the application of scRNA‐seq in fundamental research but also provide more reliable data support for tumor microenvironment studies, disease mechanism investigations, and clinical translational applications.

## 4. Materials and Methods

### 4.1. Ethic Statement

This study was conducted in accordance with the Declaration of Helsinki and approved by the Ethics Committee of Jiangsu Cancer Hospital (2023K‐009).

### 4.2. Patients

Lung cancer specimens from three lung adenocarcinoma patients were recruited from the Biobank of Jiangsu Cancer Hospital (Jiangsu Institute of Cancer Research) between December 2023 and March 2024. Two pathologists identified and reviewed all patient samples to characterize the histopathological features. Written informed consent was obtained from all patients.

### 4.3. Note on Sample Design

By dividing each patient’s tissue into two halves for parallel sequencing, we minimized spatial heterogeneity and ensured that the results between the droplet‐ and microwell‐based platforms are directly comparable.

### 4.4. Tissue Dissociation and Preparation

Fresh tissue samples were obtained from three patients, and for each patient, a single anatomical site was selected. Each tissue was immediately divided into two equal halves to ensure comparability for parallel single‐cell RNA‐seq analysis on two platforms: the droplet‐based (10x Genomics) and microwell‐based (Singleron) systems. Samples were stored in sCelLive Tissue Preservation Solution (Singleron) on ice and processed within 30 min after surgery. Tissues were washed three times with Hanks Balanced Salt Solution (HBSS), minced into small pieces, and digested with 3 mL sCelLive Tissue Dissociation Solution using the Singleron PythoN Tissue Dissociation System at 37°C for 15 min. The resulting cell suspension was filtered through a 40 μm sterile strainer. Red blood cells were removed by adding GEXSCOPE red blood cell lysis buffer (RCLB, Singleron) at a 1:2 volume ratio, incubated at room temperature for 5–8 min, followed by centrifugation at 300 × *g* at 4°C for 5 min. Cells were then resuspended in PBS, stained with Trypan Blue, and cell viability was assessed microscopically.

### 4.5. RT and Amplification and Library Construction

The prepared single‐cell suspension (2 × 10^5^ cells/mL, diluted in PBS, HyClone) was loaded onto the microwell chip of the Singleron Matrix Single Cell Processing System. Barcoded beads were then recovered from the chip, and reverse transcription was performed on the captured mRNA to synthesize cDNA, followed by PCR amplification. The amplified cDNA was subsequently fragmented and ligated with sequencing adapters. Single‐cell RNA‐seq libraries were constructed following the standard protocols of either the GEXSCOPE Single Cell RNA Library Kit (Singleron) or the Chromium Next GEM Single Cell 3ʹ Reagent Kits v3.1 (10x Genomics). Final libraries were diluted to a concentration of 4 nM, pooled, and sequenced on the Illumina NovaSeq 6000 platform with 150 bp paired‐end reads.

### 4.6. Processing of Single‐Cell RNA‐Seq Data

Raw sequencing data generated from two scRNA‐seq platforms, a droplet‐and microwell‐based platform, were processed using their respective standard pipelines. For data from droplet‐based platform, paired‐end FASTQ files were processed using Cell Ranger (v9.0.0, 10x Genomics), which performs automated QC including the removal of low‐quality reads, correction of cell barcodes and unique molecular identifiers (UMIs), and generation of gene expression matrices. In contrast, data from the microwell‐based platform were processed using the CeleScope pipeline (v2.0.7), in which raw reads were first trimmed with Cutadapt (v3.7) to remove adapter sequences and poly‐A tails, followed by extraction of cell barcodes and UMIs. Despite differences in QC and UMI handling between the two platforms, subsequent steps were consistent. For both datasets, high‐quality reads were aligned to the human reference genome GRCh38 using STAR (v2.7.11a). Gene‐level quantification was then performed using either the built‐in Cell Ranger algorithm or featureCounts (v2.0.1), resulting in digital gene expression matrices for downstream single‐cell transcriptomic analyses.

### 4.7. QC for Single‐Cell RNA‐Seq Data

For QC of scRNA‐seq data, we applied sample‐specific filtering criteria to remove low‐quality cells based on the number of detected genes per cell (nFeature_RNA), the proportion of mitochondrial gene expression (percent.mt), and the total RNA counts per cell (nCount_RNA). In the microwell‐based platform, we retained cells with 200 < nFeature_RNA < 7000, percent.mt < 25%, and nCount_RNA < 30,000. In the droplet‐based platform, we retained cells with 200 < nFeature_RNA < 8000, percent.mt < 25%, and nCount_RNA < 60,000 to maintain data quality. These filtering steps were performed using the subset function in Seurat (v5.0.1) [40] to optimize cell selection for downstream analysis.

### 4.8. Batch Correction and Clustering

We applied three widely used methods to correct batch effects in the processed single‐cell transcriptomic data to enhance the consistency and comparability across samples. The first approach employed the Liger (v2.0.1) [41] for batch correction and clustering. We applied integrative nonnegative matrix factorization (iNMF) to learn both dataset‐specific and shared factor spaces. Quantile normalization was then used to align factor loadings across datasets. The second approach utilized the Seurat package (v5.1.0) for data integration. Canonical correlation analysis (CCA) was performed to identify gene pairs with high inter‐dataset correlation, and anchor points were identified using FindIntegrationAnchors. Batch effects were removed via IntegrateData, and the integrated dataset was visualized using UMAP. The third approach involved the application of Harmony (v1.2.0) [42], an iterative clustering‐based batch correction method. Harmony first performs a modified soft k‐means clustering to group cells, calculates global and dataset‐specific centroids for each cluster, estimates correction factors based on centroid differences, and adjusts individual cells accordingly. Data integration is performed in PCA space, and batch effects are iteratively reduced. By combining these three approaches, we systematically assessed and optimized the impact of batch correction on downstream single‐cell analysis.

### 4.9. Unsupervised Clustering and Cell Type Annotation

We analyzed the single‐cell RNA‐seq dataset using the Seurat package and performed batch effect correction using Harmony. Initially, the data were normalized, followed by PCA. Subsequently, based on the Harmony‐corrected PCs, we computed UMAP embeddings and applied the unsupervised Louvain clustering algorithm to identify distinct cell populations. Starting with the coarse lineage‐specific clustering results, we iteratively performed sub‐clustering to further refine the identification of individual cell types. Finally, we annotate cells based on the marker genes of each cell type, epithelial cells (EPCAM), immune cells (PTPRC, CD3G, CD3E, and CD79A), stromal cells (PECAM1 and MME), T cells (CD3D and CD3E), B cells (CD79A, MS4A1, and CD19), epithelial cells (EPCAM, CLDN7, MUC1, CDH1, and KRT20), endothelial cells (CDH5, PECAM1, VWF, and DCN), fibroblasts (COL1A2, COL3A1, COL14A1, PDGFRB, and FGF7), myeloid cells (LYZ, CD68, CD86, ITGAM, CD163, and IL10), plasma cells (MZB1, SDC1, IGHG1), mast cells (TPSB2, TPSAB1), neutrophils (FCGR3B, CXCR2, CSF3R), cytotoxic CD8 Effector T cells (CD8A, CD8B, GZMA, GZMB, GNLY, IL2, PRF1, NKG7, IFNG, and GZMK), immunosuppressive CD4 T cells (CD4, PDCD1, HAVCR2, TOX, LAG3, TIGIT, and CTLA4), naïve T cells (CCR7, TCF7, SELL, and LEF1), NKT cells (NKG7, GNLY, FCGR3A, and NCAM1), and regulatory T cells (Tregs) (IL2RA, FOXP3, and IKZF2).

### 4.10. Processed Differential Gene Expression Analysis

To identify differentially expressed genes between cell clusters, we used the FindAllMarkers function from Seurat (v5.0.1). The analysis was performed with a logFC threshold of 0.25, and a minimum percentage of expressed cells (min.pct) set to 0.1. The logFC was used to filter out genes with minimal expression changes, while the min.pct parameter ensured that genes expressed in at least 10% of the cells within a cluster were considered. The resulting list of differentially expressed genes for each cluster was used to identify markers and further characterize the cell populations.

### 4.11. Clustering Evaluation Using Adjusted Rand Index (ARI) and Average Silhouette Width (ASW)

To assess the effectiveness of UMAP‐based clustering, we employed the ARI [43] and ASW [44]. ARI was used to quantify the similarity between clustering results and reference annotations, providing a measure of clustering accuracy. Meanwhile, ASW evaluated the compactness and separation of clusters by calculating the average silhouette score for each cell. Higher ARI and ASW scores indicate better clustering consistency and well‐separated cell populations, reflecting the robustness of batch correction and dimensionality reduction methods.

### 4.12. Cell–Cell Interaction Analysis

In this study, we utilized CellChat (v2.1.2) [45] to analyze cell–cell communication, with a focus on key computational steps. The function identifyOverExpressedGenes was employed to detect highly expressed LR genes, ensuring the inclusion of biologically relevant signaling molecules. Next, significant LR interactions were identified based on these genes, providing the foundation for downstream analyses. To enhance prediction accuracy, smoothData was applied to refine expression values using protein–protein interaction (PPI) networks. We then computed intercellular communication probabilities using computeCommunProb, allowing us to infer interaction strength between cell populations. Finally, netVisual_circle was used to visualize the number and strength of cell–cell interactions in a circular network diagram, offering insights into key signaling patterns. These analytical steps collectively facilitated a comprehensive understanding of intercellular communication dynamics.

### 4.13. Trajectory Analysis

Cell differentiation trajectory was reconstructed with Monocle2 (v2.30.1) [46]. HVGs were used to sort cells in order of spatial–temporal differentiation. We used DDRTree to perform variable feature identification and dimension‐reduction. Finally, the trajectory was visualized by the plot_cell_trajectory function.

### 4.14. Flow Cytometry

Cell samples were thawed in a 37°C water bath and collected by centrifuging at 150 rcf for 5 min. Cells were washed and resuspended in 1 mL PBS (Gibco, ThermoFisher Scientific). About 1 µL of viability dyes (biogems) was used to stain the dead cells, followed by incubating at 4°C for 30 min. Cells were washed and resuspended in 500 µL PBS. About 1 µL of PE anti‐human CD326 (Ep‐CAM) antibody (BioLegend) and APC anti‐human CD45 antibody (BioLegend) was added to stain the cells with surface markers, followed by incubating at 4°C for 30 min. Cells were washed and resuspended in PBS, then analyzed on the flow cytometer (Challenbio).

### 4.15. Statistical Analysis

All statistical analyses and data visualization were performed using R (v4.3.3). Basic data preprocessing, figure generation, and statistical tests were conducted using standard R packages. For comparison between two groups, a two‐tailed unpaired Student’s *t*‐test was applied unless otherwise specified. A *p*‐value < 0.05 was considered statistically significant. All plots were generated using ggplot2 (v3.5.1) [47], Seurat, and ComplexHeatmap (v2.18.0) [48], among other R packages.

## Author Contributions

Shuai Wang contributed to data analysis and manuscript writing. Yuxian Feng performed partial data analysis. Qiongdan Zhang conducted flow cytometry experiments. Yue Cui and Yi Qiao provided data integration, coordination, and methodological guidance. Hao Huang, Xuan Pan, and Jing Tu conceived and supervised the study and served as corresponding authors.

## Funding

The study was supported by the National Natural Science Foundation of China (Grants 62371128 and 82474598).

## Conflicts of Interest

The authors declare no conflicts of interest.

## Supporting Information

Additional supporting information can be found online in the Supporting Information section.

## Supporting information


**Supporting Information** Figure S1: PCA analysis of 3 pairs of samples from two platforms. Figure S2: Expression dot plot of marker genes. Figure S3: Flow cytometry. Figure S4: Umap plot showing the expression of Neu markers. Figure S5–6: Heatmap of genes with opposite expression patterns. Figure S7: Heatmap of genes with opposite expression patterns. Figure S8: Ligand–receptor interaction bubble plot.

## Data Availability

The data that support the findings of this study are available upon request from the corresponding author. The data are not publicly available due to privacy or ethical restrictions.

## References

[1] Tang F. , Barbacioru C. , and Wang Y. , et al.mRNA-Seq Whole-Transcriptome Analysis of a Single Cell, Nature Methods. (2009) 6, no. 5, 377–382, 10.1038/nmeth.1315, 2-s2.0-67349146589. 19349980

[2] Tang F. , Barbacioru C. , and Nordman E. , et al.RNA-Seq Analysis to Capture the Transcriptome Landscape of a Single Cell, Nature Protocols. (2010) 5, no. 3, 516–535, 10.1038/nprot.2009.236, 2-s2.0-77749323185. 20203668 PMC3847604

[3] Ramskold D. , Luo S. , and Wang Y. C. , et al.Full-Length mRNA-Seq From Single-Cell Levels of RNA and Individual Circulating Tumor Cells, Nature Biotechnology. (2012) 30, no. 8, 777–782, 10.1038/nbt.2282, 2-s2.0-84864880991. PMC3467340 22820318

[4] Hashimshony T. , Wagner F. , Sher N. , and Yanai I. , CEL-Seq: Single-Cell RNA-Seq by Multiplexed Linear Amplification, Cell Reports. (2012) 2, no. 3, 666–673, 10.1016/j.celrep.2012.08.003, 2-s2.0-84866953427. 22939981

[5] Jaitin D. A. , Kenigsberg E. , and Keren-Shaul H. , et al.Massively Parallel Single-Cell RNA-Seq for Marker-Free Decomposition of Tissues Into Cell Types, Science. (2014) 343, no. 6172, 776–779, 10.1126/science.1247651, 2-s2.0-84893905629. 24531970 PMC4412462

[6] Picelli S. , Björklund Å. K. , Faridani O. R. , Sagasser S. , Winberg G. , and Sandberg R. , Smart-Seq2 for Sensitive Full-Length Transcriptome Profiling in Single Cells, Nature Methods. (2013) 10, no. 11, 1096–1098, 10.1038/nmeth.2639, 2-s2.0-84887101406. 24056875

[7] Hashimshony T. , Senderovich N. , and Avital G. , et al.CEL-Seq2: Sensitive Highly-Multiplexed Single-Cell RNA-Seq, Genome Biology. (2016) 17, no. 1, 10.1186/s13059-016-0938-8, 2-s2.0-84964452502, 77. 27121950 PMC4848782

[8] Bagnoli J. W. , Ziegenhain C. , and Janjic A. , et al.Sensitive and Powerful Single-Cell RNA Sequencing Using mcSCRB-Seq, Nature Communications. (2018) 9, no. 1, 10.1038/s41467-018-05347-6, 2-s2.0-85050811800, 2937. PMC6062574 30050112

[9] Hong M. , Tao S. , and Zhang L. , et al.RNA Sequencing: New Technologies and Applications in Cancer Research, Journal of Hematology & Oncology. (2020) 13, no. 1, 10.1186/s13045-020-01005-x, 166. 33276803 PMC7716291

[10] Macosko E. Z. , Basu A. , and Satija R. , et al.Highly Parallel Genome-Wide Expression Profiling of Individual Cells Using Nanoliter Droplets, Cell. (2015) 161, no. 5, 1202–1214, 10.1016/j.cell.2015.05.002, 2-s2.0-84929684999. 26000488 PMC4481139

[11] Klein A. M. , Mazutis L. , and Akartuna I. , et al.Droplet Barcoding for Single-Cell Transcriptomics Applied to Embryonic Stem Cells, Cell. (2015) 161, no. 5, 1187–1201, 10.1016/j.cell.2015.04.044, 2-s2.0-84929684998. 26000487 PMC4441768

[12] Zheng G. X. , Terry J. M. , and Belgrader P. , et al.Massively Parallel Digital Transcriptional Profiling of Single Cells, Nature Communications. (2017) 8, 10.1038/ncomms14049, 2-s2.0-85009446777, 14049. PMC5241818 28091601

[13] Gierahn T. M. , Wadsworth M. H.2nd, and Hughes T. K. , et al.Seq-Well: Portable, Low-Cost RNA Sequencing of Single Cells at High Throughput, Nature Methods. (2017) 14, no. 4, 395–398, 10.1038/nmeth.4179, 2-s2.0-85012271992. 28192419 PMC5376227

[14] Han X. , Wang R. , and Zhou Y. , et al.Mapping the Mouse Cell Atlas by Microwell-Seq, Cell. (2018) 172, no. 5, 1091–1107, 10.1016/j.cell.2018.02.001, 2-s2.0-85042366842. 29474909

[15] Dura B. , Choi J. Y. , and Zhang K. , et al.scFTD-Seq: Freeze-Thaw Lysis Based, Portable Approach Toward Highly Distributed Single-Cell 3^′^ mRNA Profiling, Nucleic Acids Research. (2019) 47, no. 3. 10.1093/nar/gky1173 PMC6379653 30462277

[16] Chen G. , Xu W. , Long Z. , Chong Y. , Lin B. , and Jie Y. , Single-Cell Technologies Provide Novel Insights Into Liver Physiology and Pathology, Journal of Clinical and Translational Hepatology. (2023) 12, no. 1, 79–90, 10.14218/JCTH.2023.00224. 38250462 PMC10794276

[17] Ye W. , Lian Q. , Ye C. , and Wu X. , A Survey on Methods for Predicting Polyadenylation Sites From DNA Sequences, Bulk RNA-Seq, and Single-Cell RNA-Seq, Genomics, Proteomics & Bioinformatics. (2023) 21, no. 1, 67–83, 10.1016/j.gpb.2022.09.005. PMC10372920 36167284

[18] Liu D. , Sun M. , and Zhang J. , et al.Single-Cell Droplet Microfluidics for Biomedical Applications, The Analyst. (2022) 147, no. 11, 2294–2316, 10.1039/D1AN02321G. 35506869

[19] Park J. Y. , Morgan M. , and Sachs A. N. , et al.Single Cell Trapping in Larger Microwells Capable of Supporting Cell SpReading and Proliferation, Microfluidics and Nanofluidics. (2010) 8, no. 2, 263–268, 10.1007/s10404-009-0503-9, 2-s2.0-77649233856. 20352022 PMC2845479

[20] Lai R. L. and Huang N.-T. , Dimensional Analysis and Parametric Studies of the Microwell for Particle Trapping, Microfluidics and Nanofluidics. (2019) 23, no. 11, 10.1007/s10404-019-2289-8, 2-s2.0-85073422960, 121.

[21] Pan Y. , Cao W. , Mu Y. , and Zhu Q. , Microfluidics Facilitates the Development of Single-Cell RNA Sequencing, Biosensors. (2022) 12, no. 7, 10.3390/bios12070450, 450. 35884253 PMC9312765

[22] Han X. , Xu X. , Yang C. , and Liu G. , Microfluidic Design in Single-Cell Sequencing and Application to Cancer Precision Medicine, Cell Reports Methods. (2023) 3, no. 9, 10.1016/j.crmeth.2023.100591, 100591. 37725985 PMC10545941

[23] Chang J.-T. , Liu L.-B. , Wang P.-G. , and An J. , Single-Cell RNA Sequencing to Understand Host-Virus Interactions, Virologica Sinica. (2024) 39, no. 1, 1–8, 10.1016/j.virs.2023.11.009. 38008383 PMC10877424

[24] Mereu E. , Lafzi A. , and Moutinho C. , et al.Benchmarking Single-Cell RNA-Sequencing Protocols for Cell Atlas Projects, Nature Biotechnology. (2020) 38, no. 6, 747–755, 10.1038/s41587-020-0469-4. 32518403

[25] Chen W. , Zhao Y. , and Chen X. , et al.A Multicenter Study Benchmarking Single-Cell RNA Sequencing Technologies Using Reference Samples, Nature Biotechnology. (2021) 39, no. 9, 1103–1114, 10.1038/s41587-020-00748-9. PMC11245320 33349700

[26] Wang X. , He Y. , Zhang Q. , Ren X. , and Zhang Z. , Direct Comparative Analyses of 10X Genomics Chromium and Smart-Seq2, Genomics, Proteomics & Bioinformatics. (2021) 19, no. 2, 253–266, 10.1016/j.gpb.2020.02.005. PMC8602399 33662621

[27] Gao C. , Zhang M. , and Chen L. , The Comparison of Two Single-Cell Sequencing Platforms: BD Rhapsody and 10x Genomics Chromium, Current Genomics. (2020) 21, no. 8, 602–609, 10.2174/1389202921999200625220812. 33414681 PMC7770630

[28] Ilicic T. , Kim J. K. , and Kolodziejczyk A. A. , et al.Classification of Low Quality Cells From Single-Cell RNA-Seq Data, Genome Biology. (2016) 17, no. 1, 10.1186/s13059-016-0888-1, 2-s2.0-84958058589, 29. 26887813 PMC4758103

[29] Osorio D. , Cai J. J. , and Mathelier A. , Systematic Determination of the Mitochondrial Proportion in Human and Mice Tissues for Single-Cell RNA-Sequencing Data Quality Control, Bioinformatics. (2021) 37, no. 7, 963–967, 10.1093/bioinformatics/btaa751. 32840568 PMC8599307

[30] Laughney A. M. , Hu J. , and Campbell N. R. , et al.Regenerative Lineages and Immune-Mediated Pruning in Lung Cancer Metastasis, Nature Medicine. (2020) 26, no. 2, 259–269, 10.1038/s41591-019-0750-6. PMC7021003 32042191

[31] He Y. , Yu F. , and Tian Y. , et al.Single-Cell RNA Sequencing Unravels Distinct Tumor Microenvironment of Different Components of Lung Adenocarcinoma Featured as Mixed Ground-Glass Opacity, Frontiers in Immunology. (2022) 13, 10.3389/fimmu.2022.903513, 903513. 35874770 PMC9299373

[32] Renaut S. , Saavedra Armero V. , and Boudreau D. K. , et al.Single-Cell and Single-Nucleus RNA-Sequencing From Paired Normal-Adenocarcinoma Lung Samples Provide Both Common and Discordant Biological Insights, PLoS Genetics. (2024) 20, no. 5, 10.1371/journal.pgen.1011301. PMC11166281 38814983

[33] Wang Z. , Yang L. , and Wang W. , et al.Comparative Immunological Landscape Between Pre- and Early-Stage LUAD Manifested as Ground-Glass Nodules Revealed by scRNA and scTCR Integrated Analysis, Cell Communication and Signaling. (2023) 21, no. 1, 10.1186/s12964-023-01322-x, 325. 37957625 PMC10644515

[34] Ding J. , Adiconis X. , and Simmons S. K. , et al.Systematic Comparison of Single-Cell and Single-Nucleus RNA-Sequencing Methods, Nature Biotechnology. (2020) 38, no. 6, 737–746, 10.1038/s41587-020-0465-8. PMC7289686 32341560

[35] Salcher S. , Sturm G. , and Horvath L. , et al.High-Resolution Single-Cell Atlas Reveals Diversity and Plasticity of Tissue-Resident Neutrophils in Non-Small Cell Lung Cancer, Cancer Cell. (2022) 40, no. 12, 1503–1520, 10.1016/j.ccell.2022.10.008. 36368318 PMC9767679

[36] Wang L. , Liu Y. , and Dai Y. , et al.Single-Cell RNA-Seq Analysis Reveals BHLHE40-Driven pro-Tumour Neutrophils With Hyperactivated Glycolysis in Pancreatic Tumour Microenvironment, Gut. (2023) 72, no. 5, 958–971, 10.1136/gutjnl-2021-326070. 35688610 PMC10086491

[37] Miyake K. , Ito J. , Nakabayashi J. , Shichino S. , Ishiwata K. , and Karasuyama H. , Single Cell Transcriptomics Clarifies the Basophil Differentiation Trajectory and Identifies Pre-Basophils Upstream of Mature Basophils, Nature Communications. (2023) 14, no. 1, 10.1038/s41467-023-38356-1, 2694. PMC10195816 37202383

[38] Colino-Sanguino Y. , Rodriguez de la Fuente L. , and Gloss B. , et al.Performance Comparison of High Throughput Single-Cell RNA-Seq Platforms in Complex Tissues, Heliyon. (2024) 10, no. 17, 10.1016/j.heliyon.2024.e37185. PMC11408078 39296129

[39] Salcher S. , Heidegger I. , and Untergasser G. , et al.Comparative Analysis of 10X Chromium vs. BD Rhapsody Whole Transcriptome Single-Cell Sequencing Technologies in Complex Human Tissues, Heliyon. (2024) 10, no. 7, 10.1016/j.heliyon.2024.e28358. PMC11059509 38689972

[40] Hao Y. , Stuart T. , and Kowalski M. H. , et al.Dictionary Learning for Integrative, Multimodal and Scalable Single-Cell Analysis, Nature Biotechnology. (2024) 42, no. 2, 293–304, 10.1038/s41587-023-01767-y. PMC10928517 37231261

[41] Liu J. , Gao C. , Sodicoff J. , Kozareva V. , Macosko E. Z. , and Welch J. D. , Jointly Defining Cell Types from Multiple Single-Cell Datasets Using LIGER, Nature Protocols. (2020) 15, no. 11, 3632–3662, 10.1038/s41596-020-0391-8. 33046898 PMC8132955

[42] Korsunsky I. , Millard N. , and Fan J. , et al.Fast, Sensitive and Accurate Integration of Single-Cell Data With Harmony, Nature Methods. (2019) 16, no. 12, 1289–1296, 10.1038/s41592-019-0619-0. 31740819 PMC6884693

[43] Hubert L. and Arabie P. , Comparing Partitions, Journal of Classification. (1985) 2, no. 1, 193–218, 10.1007/BF01908075, 2-s2.0-0000008146.

[44] Lotfollahi M. , Wolf F. A. , and Theis F. J. , Generative Modeling and Latent Space Arithmetics Predict Single-Cell Perturbation Response Across Cell Types, Studies and Species, 2018, BioRxiv.

[45] Jin S. , Guerrero-Juarez C. F. , and Zhang L. , et al.Inference and Analysis of Cell–Cell Communication Using CellChat, Nature Communications. (2021) 12, no. 1, 10.1038/s41467-021-21246-9, 1088. PMC7889871 33597522

[46] Trapnell C. , Cacchiarelli D. , and Grimsby J. , et al.The Dynamics and Regulators of Cell Fate Decisions Are Revealed by Pseudotemporal Ordering of Single Cells, Nature Biotechnology. (2014) 32, no. 4, 381–386, 10.1038/nbt.2859, 2-s2.0-84900873950. PMC4122333 24658644

[47] Ginestet C. , ggplot2: Elegant Graphics for Data Analysis, Journal of the Royal Statistical Society Series A: Statistics in Society. (2011) 174, no. 1, 245–246, 10.1111/j.1467-985X.2010.00676_9.x.

[48] Gu Z. , Complex Heatmap Visualization, iMeta. (2022) 1, no. 3. 10.1002/imt2.43 PMC10989952 38868715

