# Supplementary material for: Systematic Comparison of Droplet‐Based and Microwell‐Based Platforms for Comprehensive Single‐Cell Transcriptomic Analysis in Clinical Samples
Source: IET Nanobiotechnol. 2026 Feb 25;2026:9314222. doi: 10.1049/nbt2/9314222 (PMC12933413; doi:10.1049/nbt2/9314222)
Supplement: Supplementary file 1 — Supporting Information Figure S1: PCA analysis of 3 pairs of samples from two platforms. Figure S2: Expression dot plot of marker genes. Figure S3: Flow cytometry. Figure S4: Umap plot showing the expression of Neu markers. Figure S5–6: Heatmap of genes with opposite expression patterns. Figure S7: Heatmap of genes with opposite expression patterns. Figure S8: Ligand–receptor interaction bubble plot. [file NBT2-2026-9314222-s001.docx]

## Supporting Information

Supporting Information containing 7 figures is available online.


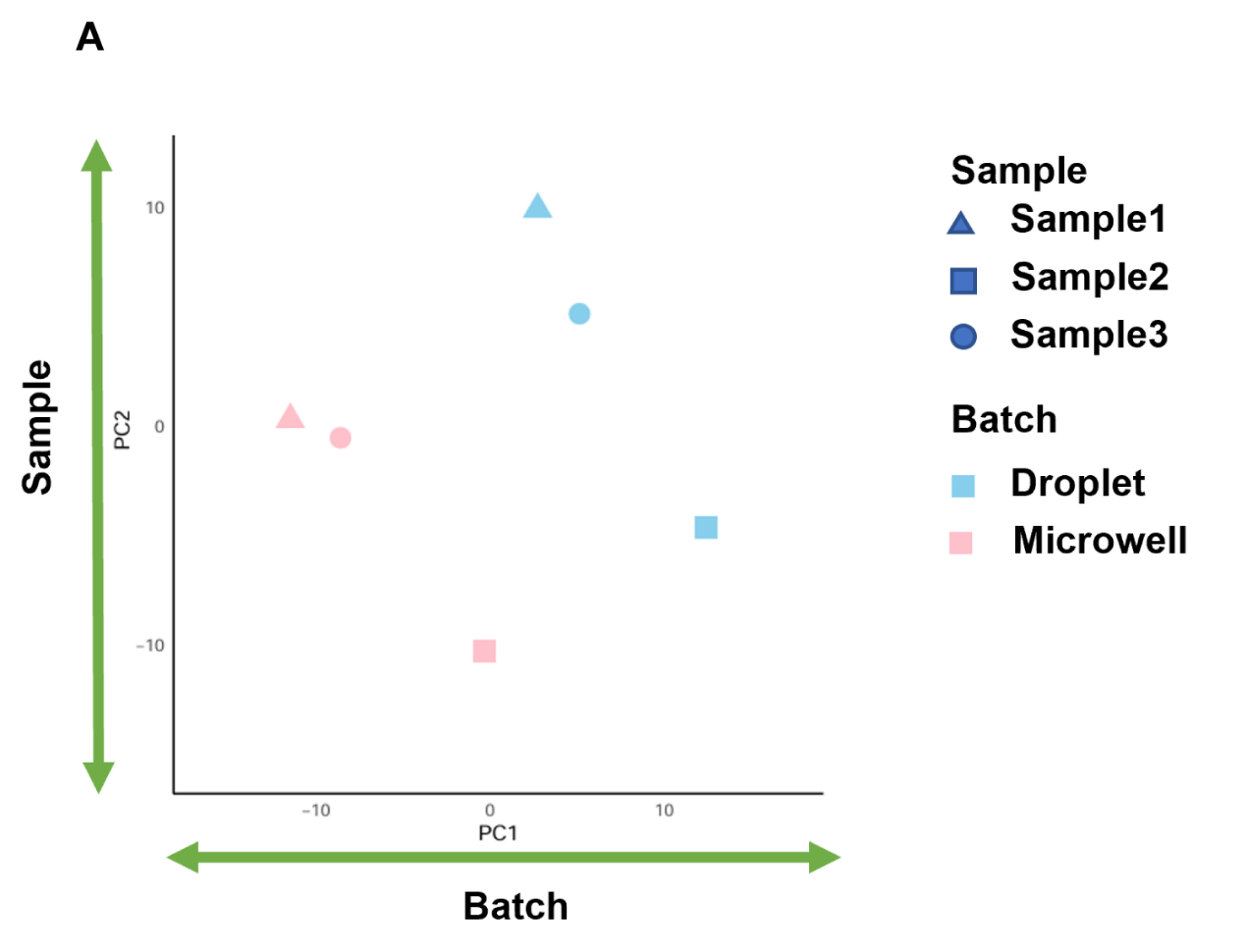


**Figure S1.** PCA analysis of 3 pairs of samples from two platforms.


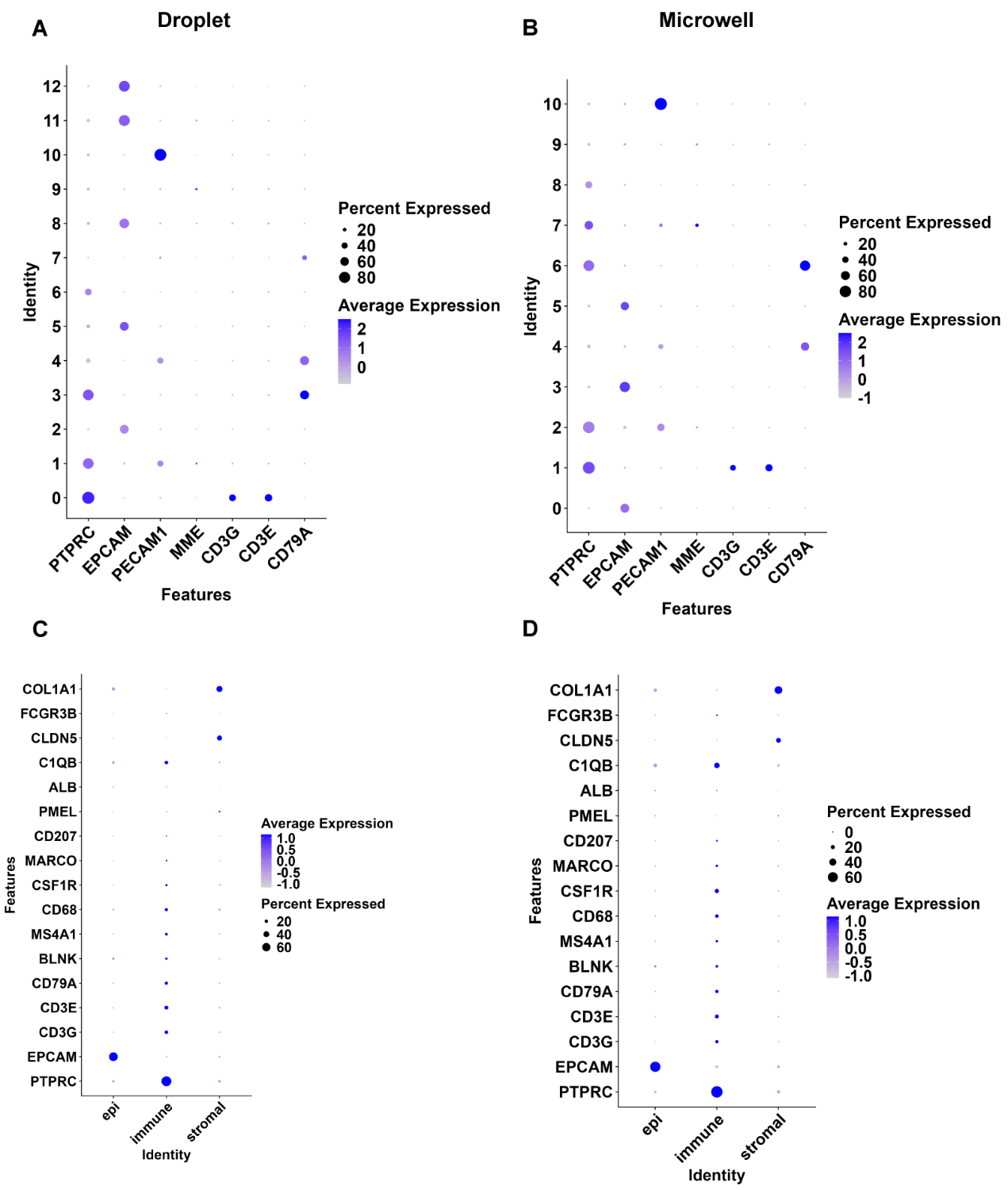


**Figure S2**. (A, B) Dot plot showing the expression of marker genes across seurat clusters. (C, D) Dot plot showing the expression of marker genes across three major cell types.


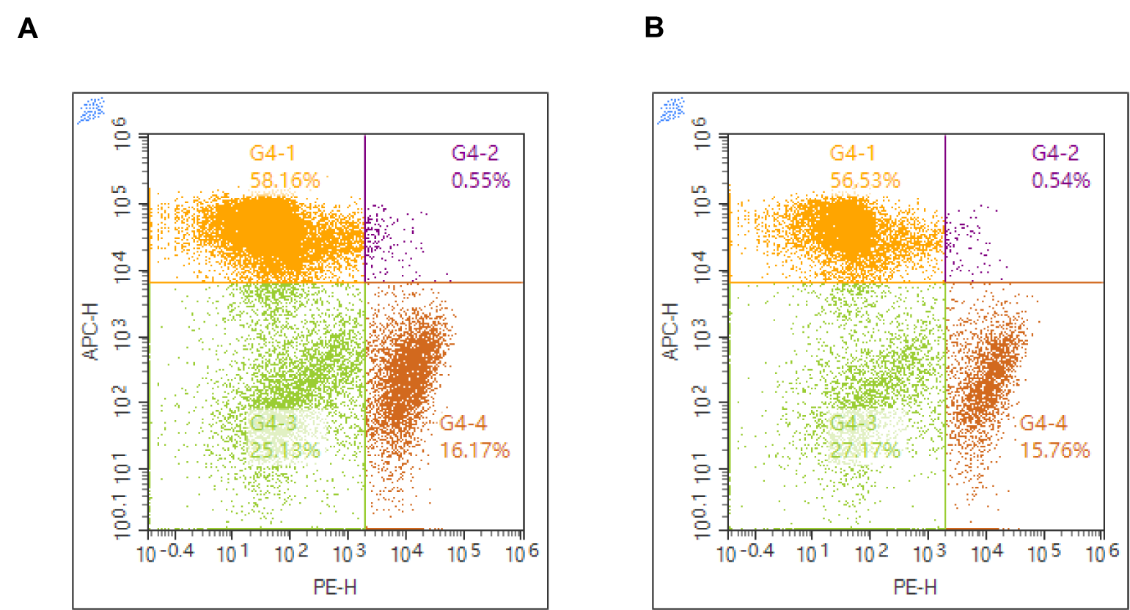


**Figure S3**. (A, B) Figures S3A and S3B show parallel flow cytometry of retained samples from one patient, yielding similar results for validation. The x-axis indicates epithelial markers, the y-axis immune markers, and the upper right quadrant shows the proportion of immune cells.


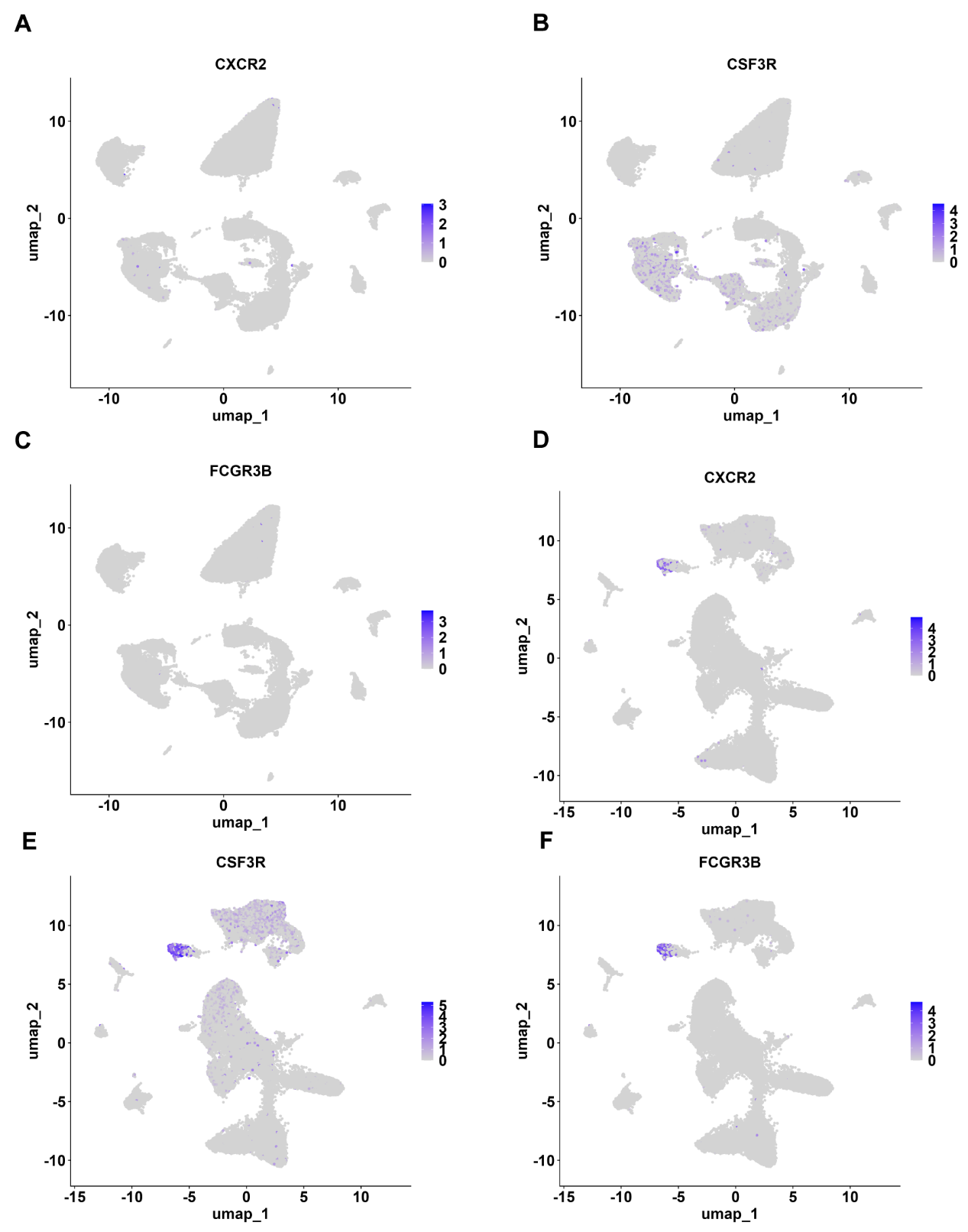


**Figure S4**. (A, C) Umap plot showing the expression of CXCR2, CSF3R, FCGR3B on the droplet-based platform. (D, F) Umap plot showing the expression of CXCR2, CSF3R, FCGR3B on the microwell-based platform.


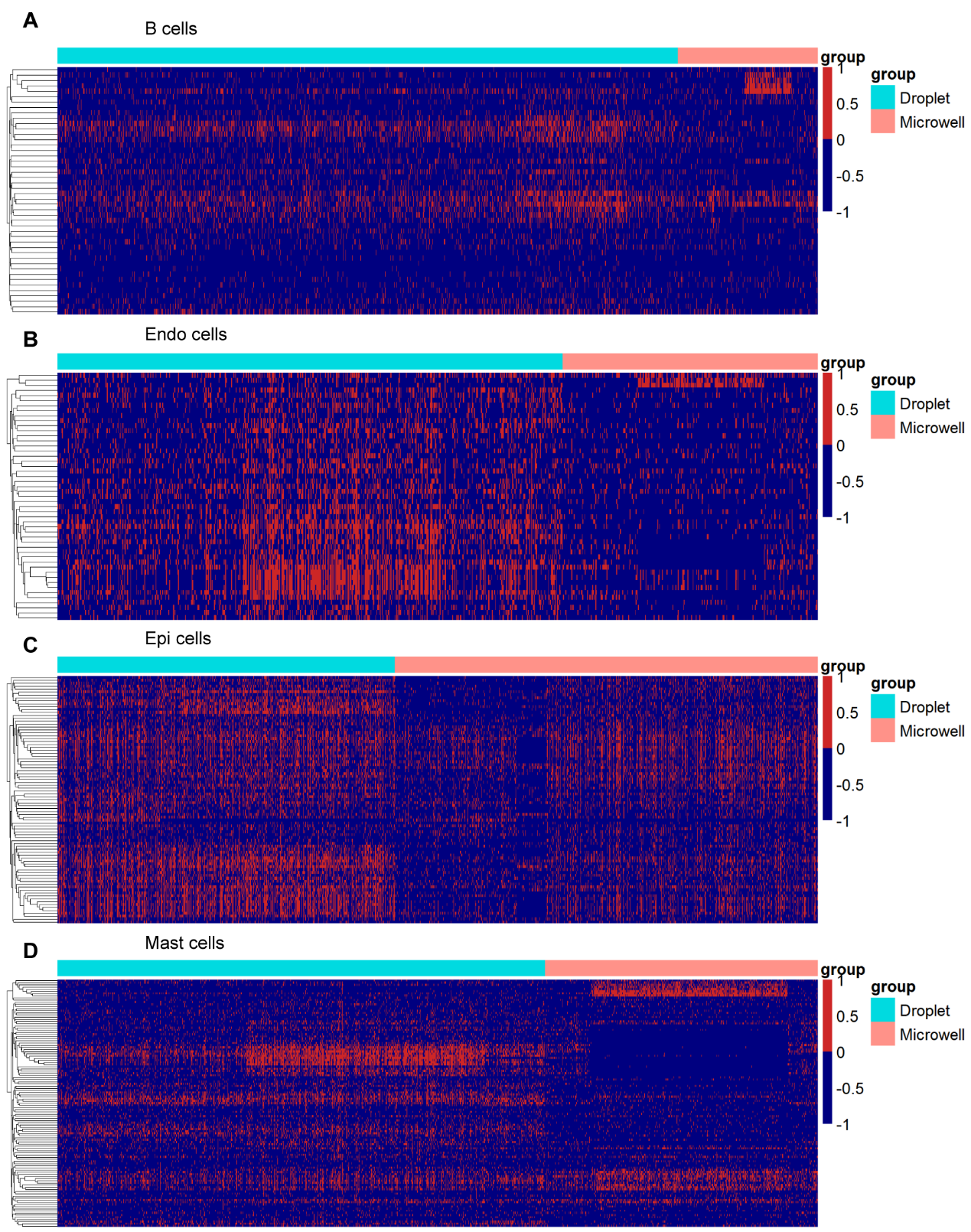


**Figure S5**. (A, B, C, D) Heatmap of genes with opposite expression patterns in B cells, Endothelial cells, Epithelial cells, Mast cells on two platforms.


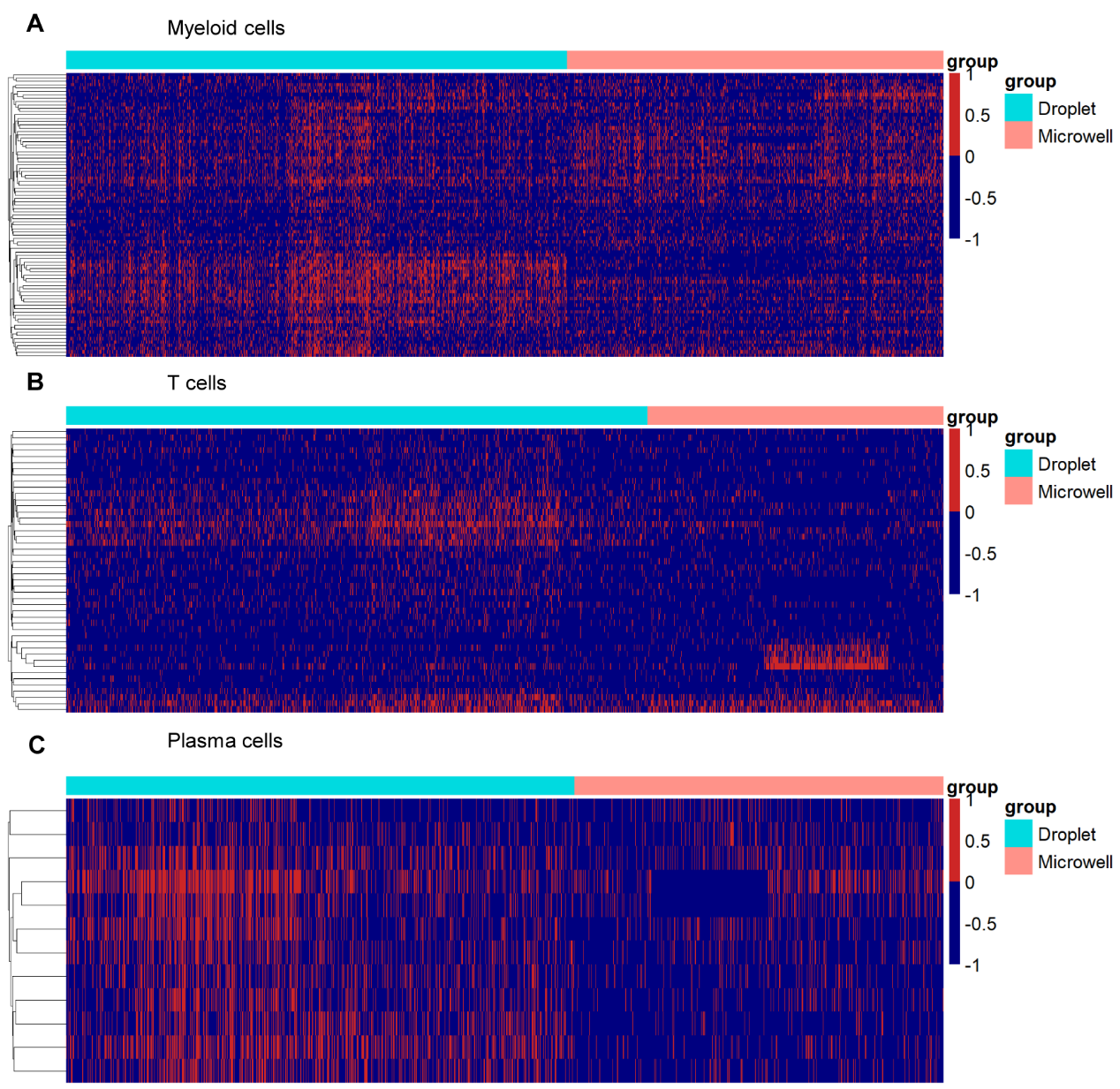


**Figure S6**. (A, B, C) Heatmap of genes with opposite expression patterns in Myeloid cells, T cells and Plasma cells on two platforms.


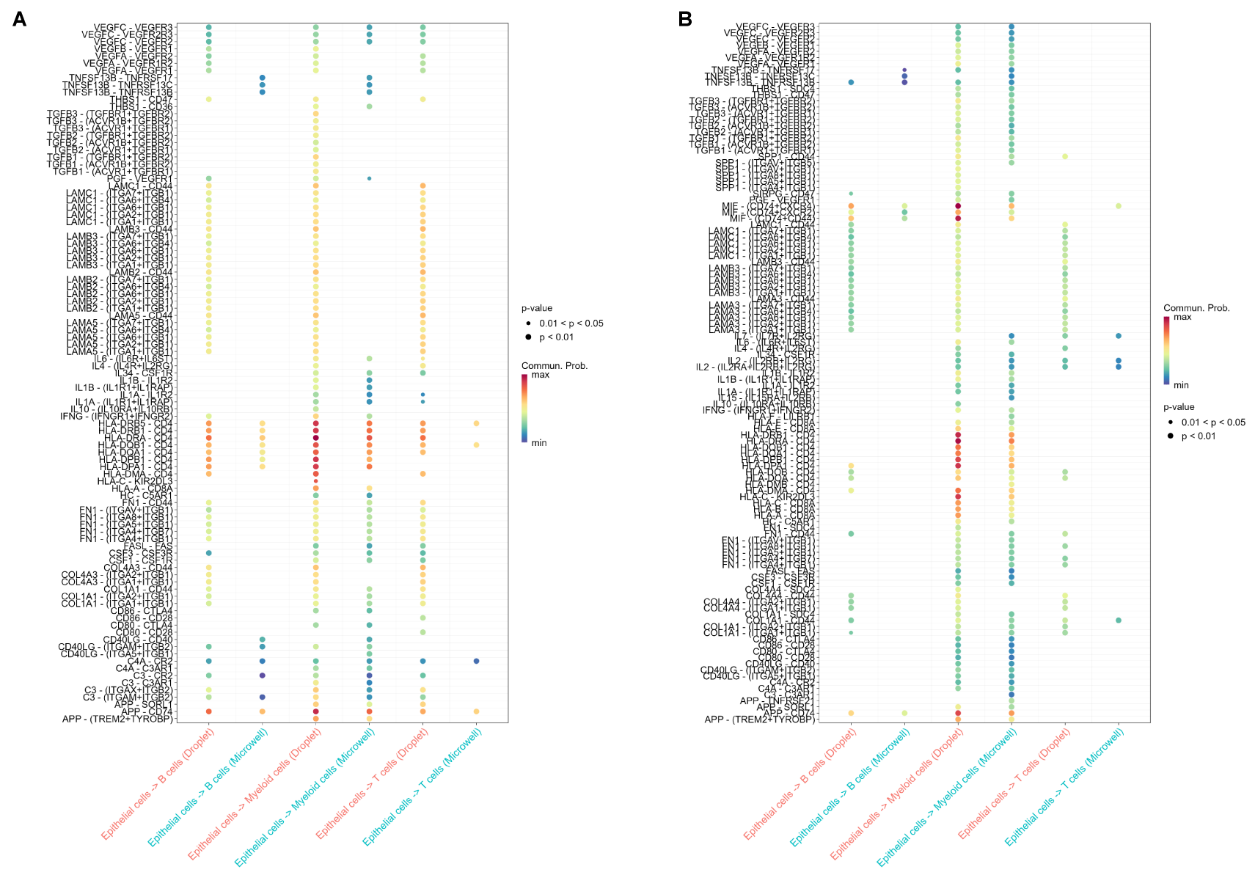


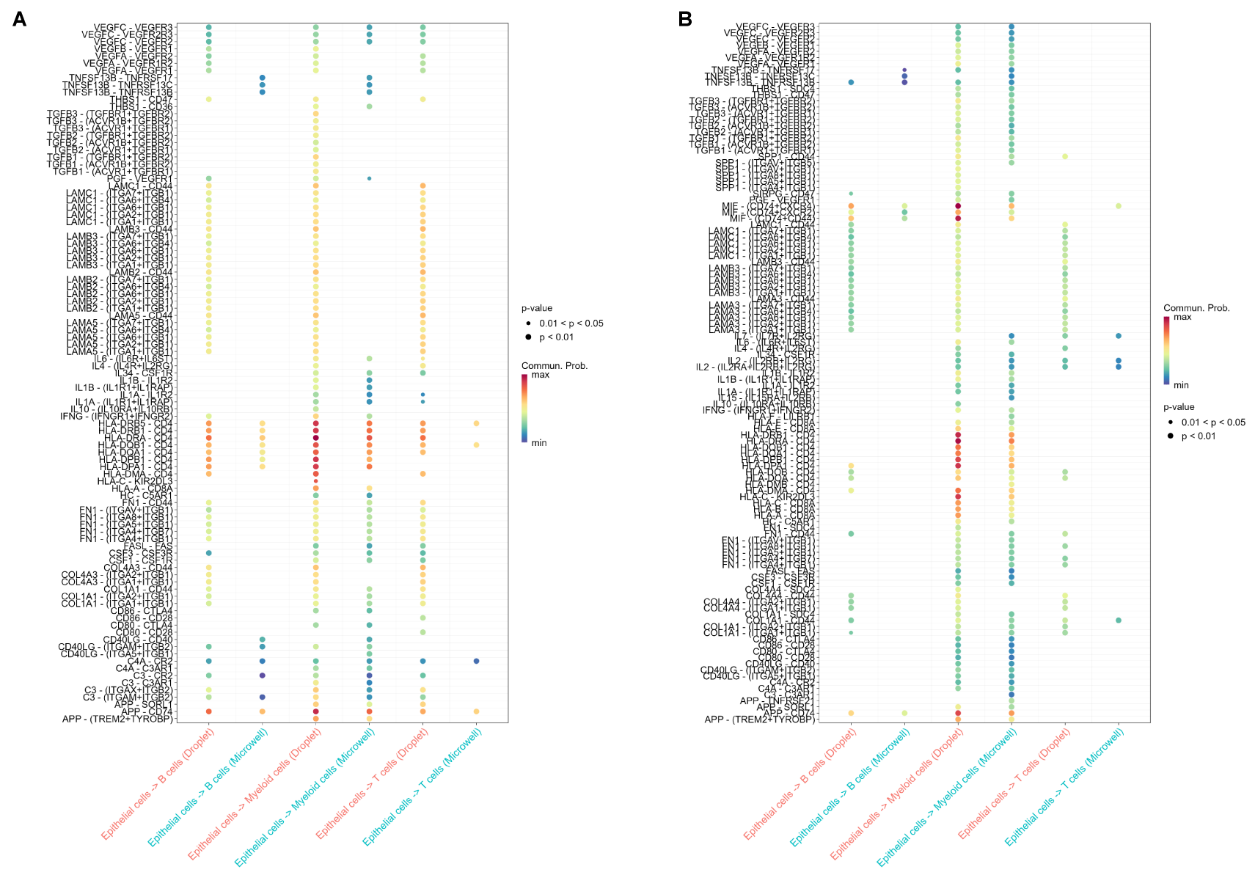


**Figure S7**. (A) Ligand-receptor interaction bubble plot for patient 2. (B) Ligand-receptor interaction bubble plot for patient 3.


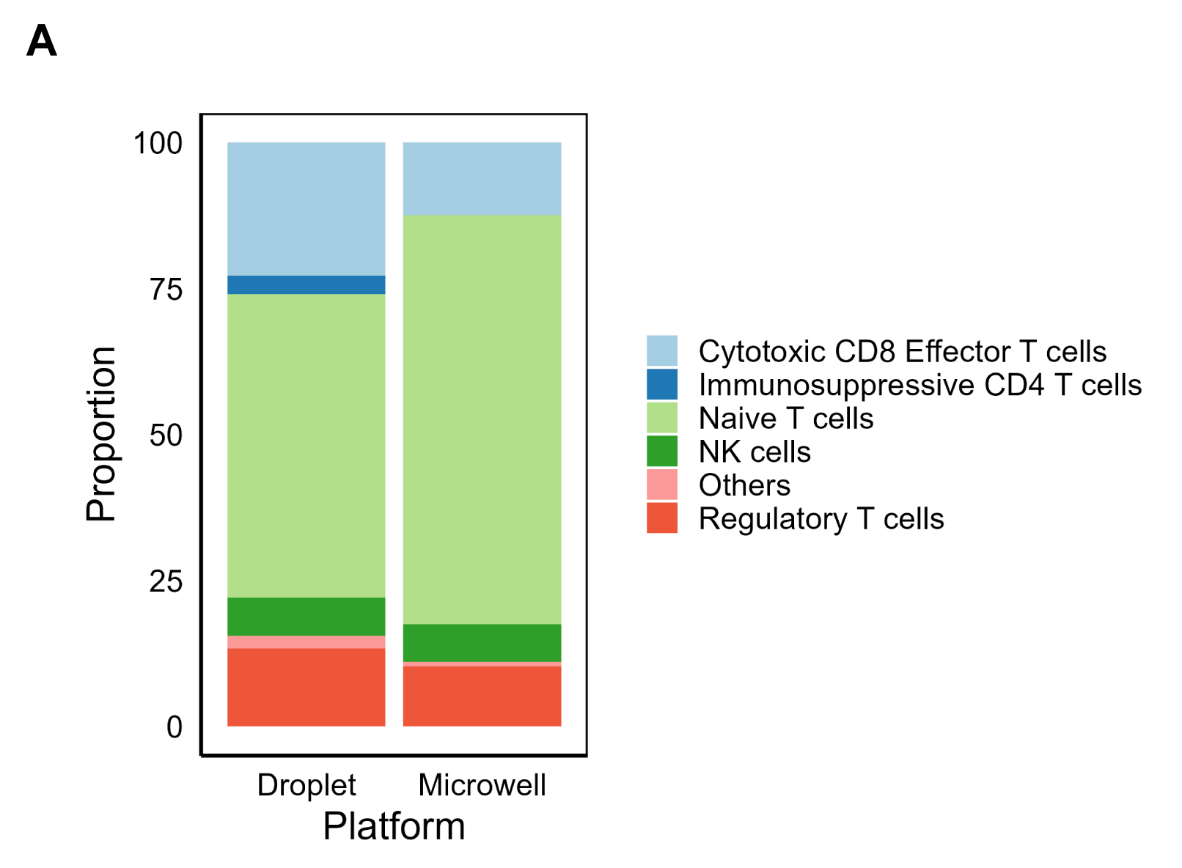


**Figure S8**. Proportion of T subtypes in two platforms.
